# Arabidopsis Target of Rapamycin Coordinates With Transcriptional and Epigenetic Machinery to Regulate Thermotolerance

**DOI:** 10.3389/fpls.2021.741965

**Published:** 2021-10-28

**Authors:** Mohan Sharma, Muhammed Jamsheer K., Brihaspati Narayan Shukla, Manvi Sharma, Prakhar Awasthi, Sanjeet Kumar Mahtha, Gitanjali Yadav, Ashverya Laxmi

**Affiliations:** National Institute of Plant Genome Research, New Delhi, India

**Keywords:** glucose, TOR-E2Fa signalling, heat stress, gene regulation, epigenetics, climate change

## Abstract

Global warming exhibits profound effects on plant fitness and productivity. To withstand stress, plants sacrifice their growth and activate protective stress responses for ensuring survival. However, the switch between growth and stress is largely elusive. In the past decade, the role of the target of rapamycin (TOR) linking energy and stress signalling is emerging. Here, we have identified an important role of Glucose (Glc)-TOR signalling in plant adaptation to heat stress (HS). Glc *via* TOR governs the transcriptome reprogramming of a large number of genes involved in heat stress protection. Downstream to Glc-TOR, the E2Fa signalling module regulates the transcription of heat shock factors through direct recruitment of E2Fa onto their promoter regions. Also, Glc epigenetically regulates the transcription of core HS signalling genes in a TOR-dependent manner. TOR acts in concert with p300/CREB HISTONE ACETYLTRANSFERASE1 (HAC1) and dictates the epigenetic landscape of HS loci to regulate thermotolerance. Arabidopsis plants defective in TOR and HAC1 exhibited reduced thermotolerance with a decrease in the expression of core HS signalling genes. Together, our findings reveal a mechanistic framework in which Glc-TOR signalling through different modules integrates stress and energy signalling to regulate thermotolerance.

## Introduction

Plants are immobile and are unable to escape the harmful conditions in the environment. Rather, throughout evolution, they have evolved adaptive strategies to gauge and respond to stressful conditions for survival and reproductive success. At the time of stress, plants sacrifice their growth to activate stress machinery and ensure survival. Once the stress signal is receded, plants quickly downregulate the stress machinery and activate stress recovery and growth machinery. Sugars produced through photosynthesis are pivotal to plant growth and development. Global transcriptome profiling in response to sugars or mutants defective in sugar signalling (such as TOR mutants) showed alteration in stress-induced gene transcripts such as heat shock proteins (Mishra et al., 2009; Xiong et al., 2013; Gupta et al., 2015b). TOR is an evolutionarily conserved ser/thr kinase, which works in a multiprotein complex and is a central regulator of different environmental responses in eukaryotes (Loewith and Hall, 2011; Laplante and Sabatini, 2012). The complex domain architecture of Arabidopsis TOR is evolutionarily conserved among eukaryotes, including mammals and plants (Menand et al., 2002; Zoncu et al., 2011). TOR transduces photosynthesis-derived Glc/energy signals to activate progenitor stem cells in the root and shoot apical meristem (RAM and SAM). TOR through the interaction and direct phosphorylation activates E2Fa/b transcription factors in controlling RAM and SAM (Xiong et al., 2013; Li et al., 2017). E2Fs, along with their dimerization partner (DP), control the transcription of genes underlying DNA replication, cell cycle, and stress responses through the binding on conserved *cis-*acting TTTCCCGCC and other similar elements in their promoter region (Vandepoele et al., 2005; Xiong et al., 2013; Sharma et al., 2019).

In the past years, the target of rapamycin has emerged as a central regulator of nutrient and stress responses in plants (Xiong and Sheen, 2014; Wu et al., 2019; Fu et al., 2020). TOR overexpressing (OE) plants showed longer primary roots under excessive nitrate. Moreover, plants with reduced TOR levels showed shorter primary roots with increased sensitivity to osmotic stress (Deprost et al., 2007). Cold exposure caused the transient inhibition of TOR kinase activity and converted the purple hypocotyl of *torRNAi* lines into green, which may be due to constitutive decreasing translation in *torRNAi* lines (Wang et al., 2016). Under non-stress conditions, TOR kinase phosphorylates ABA receptor PYLs, which dissociates the link between ABA and PP2C phosphatase, conferring inactivation of SnRK2. The onset of stress leads to phosphorylation of RAPTOR1 through ABA-activated SnRK2, which disrupts TOR complex association and, therefore, inhibits TOR kinase activity. Plants employ this phospho-regulatory loop to adjust growth and stress responses according to environmental conditions (Wang et al., 2018). In addition to abiotic stresses, TOR negatively regulates the defence response to bacterial and fungal pathogens through an antagonistic interaction with jasmonic and salicylic acid signalling (De Vleesschauwer et al., 2017). Among other stresses, temperature stress is a major factor that limits plant growth and productivity. However, the role of TOR in controlling temperature responses is, hitherto, unknown.

Chromatin structures lie at the core of stress responses and play crucial roles, ranging from sensory to downstream gene activation (Kumar and Wigge, 2010; Lämke and Bäurle, 2017). The structure of chromatin is remodelled at various levels, such as alternation in histone variants, nucleosome positioning, gene looping, and posttranscriptional histone modifications (Deal and Henikoff, 2010; Luo et al., 2012; Struhl and Segal, 2013). In most cases, histone acetylation correlates with transcriptional activation, whereas histone methylation can activate or repress transcription, depending on the nature and position of lysine residues that are methylated (Hu et al., 2015; Lämke et al., 2016; Liu et al., 2019; Sharma et al., 2019). Previous reports suggested the role of several chromatin remodelers and histone-modifying enzymes in the regulation of abiotic stress tolerance (Singh et al., 2014; Weng et al., 2014; Hu et al., 2015; Zheng et al., 2019; Ueda and Seki, 2020; Vlachonasios et al., 2021). The enzymes HISTONE ACETYLTRANSFERASES (HATs) catalyse the acetylation of specific lysine residues (such as K9, K14, K18, K23, and K27) in the amino-terminal tail of histones, which are associated with the transcriptional activation of genes involved in different biological processes (Boycheva et al., 2014; Shen et al., 2015).

Glc through transcription activation of HIKESHI LIKE PROTEIN1 (HLP1) regulates the expression of thermotolerance-associated HSP genes. Also, plants overexpressing TOR showed increased thermotolerance (Sharma et al., 2019). However, the underlying mechanism of how TOR controls thermotolerance response is unknown. Here, using genome-wide transcription analysis, we show that Glc governs the global transcriptome reprogramming of genes involved in heat stress. Arabidopsis seedlings challenged with heat under Glc sufficiency exhibited synergistic interaction of Glc/heat transcriptomic data with that of TOR targets. At the downstream, TOR target E2Fa occupies the promoters of *HSFA1* and *HSFA2*, which are master regulators of thermotolerance response, and regulates their transcription. Besides transcriptional control, Glc *via* TOR epigenetically modulates the promoters of thermotolerance-associated HSP genes. TOR acts in concert with HAC1 and modifies the acetylation of HS loci in conferring thermotolerance response.

## Results

### Glucose Is Essential for Plant Survival Under Extreme Heat Stress

**FIGURE 1:**
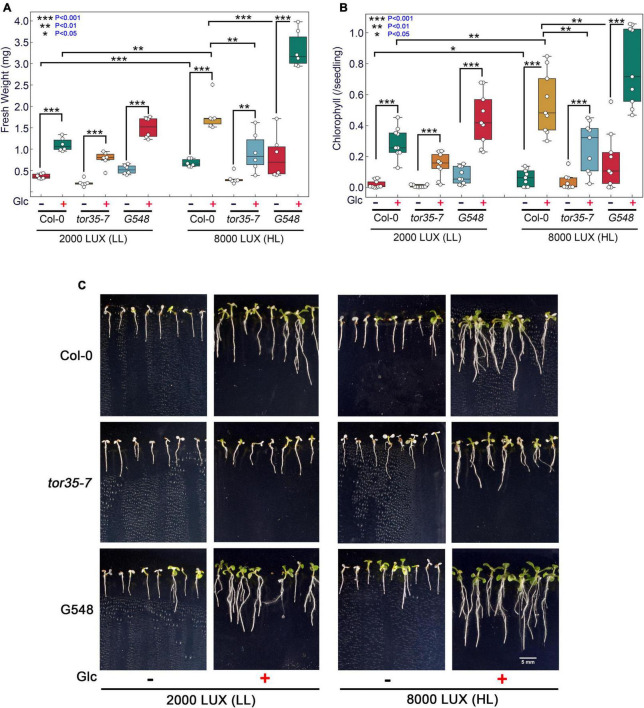
Increasing light intensity mimics the Glc-TOR-mediated thermotolerance response. **(A,B)** Measurement of fresh weight and Chl content of Arabidopsis Col-0, *tor35-7* RNAi and TOR overexpression (G548) line grown under low (2000 LUX; LL) and high (8000 LUX; HL) light intensities without or with Glc (90 mM) and after HS. **(C)** HS phenotype of Col-0, *tor35-7*, and G548 grown under LL and HL. Arabidopsis seedlings were grown directly onto 0.5X MS medium containing without or with Glc (90 mM) Glc for 7 days under LL and HL intensities and then transferred to HS. HS was applied as 1 h_37°C, 2 h_22°C, and 2.5 h_45°C, followed by recovery at 3–7 days_22°C under normal light (5000 LUX; NL) intensity. Data shown are representative of three biological replicates, each containing two (FW) and three (Chl) technical replicates with at least 20 seedlings. Experiments were independently repeated three times (*n* = 3) with similar results. (*** denote statistical differences at *p* < 0.001, ** at *p* < 0.01, and * at *p* < 0.05 as assessed by one-way ANOVA and Tukey’s HSD *post hoc* test).

Glc is a vital source of energy for most of the organisms on earth. It serves as a fundamental metabolic and signalling molecule in regulating multifaceted roles, including transcription, translation, cell cycle, and stress responses. Recently, the role of Glc-mediated TOR-E2Fa signalling in thermotolerance has been proposed (Sharma et al., 2019). However, the molecular mechanism of how Glc-driven TOR signalling governs thermotolerance is, hitherto, unknown. Under the natural environment, Glc production depends on the availability of light. These photosynthetically generated sugars could modulate root architecture, and endogenous sugar levels are regulated by fluctuating light conditions (Kircher and Schopfer, 2012; Gupta et al., 2015a). To test whether high light (HL) could provide thermotolerance to Arabidopsis plants, we grew Arabidopsis seeds on 0.5X MS media containing no or moderate Glc for 7 days under low light (LL; 2000 LUX) and HL (8000 LUX) and analysed the effect of HS. HS was applied as 1 h at 37°C, 2 h at 22°C, 2.5 h at 45°C, and 3–7 days at 22°C under normal light (NL; 5000 lux). We observed that Col-0 seedlings grown under LL and without Glc were very weak and could not recover after heat stress. However, seedlings grown under HL without Glc showed more improved stress recovery than their LL without Glc counterparts. Also, seedlings grown under LL with Glc showed better growth and improved stress recovery. Interestingly, seedlings grown under HL together with Glc showed the highest rate of stress recovery and increased thermotolerance (Figures 1A–C). Next, we assessed the phenotype of Arabidopsis Col-0, *tor35-7* RNAi, and TOR OE (G548) lines under the same experimental set-up. TOR OE survived better whereas *tor35-7* exhibited more severe death phenotypes and defects in stress recovery under all the conditions (i.e., + Glc, LL; −Glc, HL, and + Glc, HL) than Col-0 plants (Figures 1A–C). Since photosynthesis produces sucrose that is converted to Glc and fructose before entering metabolism, it was important to test whether HL-mediated thermo-protection was specific to Glc. To test this, we blocked Glc signalling/metabolism by using Glc analogue 2-deoxy-glucose (2-DG) and checked thermotolerance response under HL. 2-DG is actively transported through hexose transporters and phosphorylated but cannot be metabolised. 2-DG-6-phosphate interferes with Glc metabolism through inhibition of glycolytic enzymes (Ralser et al., 2008). Interestingly, we found that plants treated with 2-DG could not provide tolerance under HL, further substantiating the specific role of photosynthesis-derived Glc signalling in governing thermotolerance (Supplementary Figures 1A–F).

### The Presence of Glc Profoundly Alters the Expression Profile of HS-Regulated Genes

To dissect the contribution of Glc/energy in HS transcriptional response, a whole genome microarray analysis of Arabidopsis Col-0 seedlings was performed. Five-day-old Arabidopsis seedlings were subjected to Glc-free (−Glc) MS media for 24 h, followed by 24-h Glc treatment (+ Glc; 167 mM). Following acclimation to Glc medium, seedlings were transferred to HS (37°C_3 h). The cDNA was prepared from RNA isolated from Glc- and HS-treated samples and was hybridised onto Affymetrix Arabidopsis whole genome ATH1 arrays. The extent and the overlap of Glc- and HS-regulated genes at the transcriptional level were analysed to determine the unique and overlapping set of genes, which are crucial targets of Glc-HS signalling crosstalk. We used four treatment conditions and designated the following terms for Glc-free (−Glc), Glc only (+Glc), HS only (−Glc/ + HS), Glc, and HS (+ Glc/ + HS). We considered the differential expression of all HS genes affected 1.5-fold or more in the presence of Glc.

**FIGURE 2:**
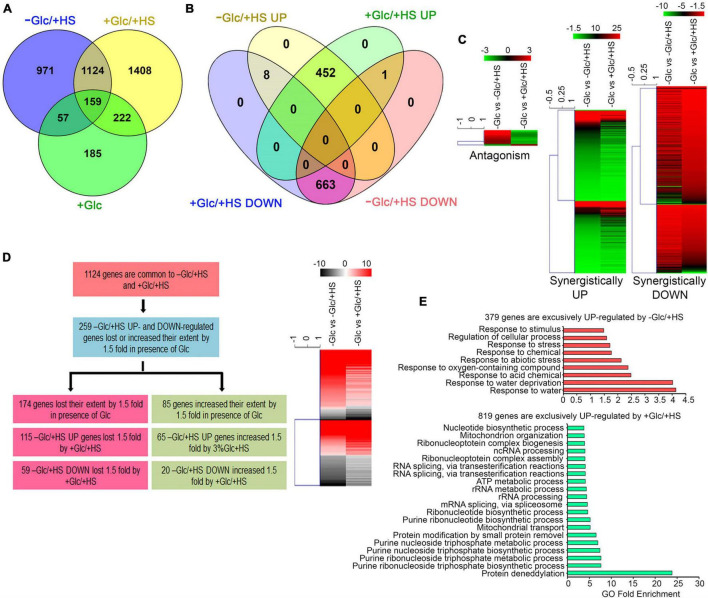
Glc triggers transcriptome reprogramming of HS-regulated genes. **(A)** A Venn diagram showing overlap of genes induced by –Glc/ + HS, + Glc/ + HS, and + Glc only. **(B,C)** A Venn diagram and heat maps showing synergistic and antagonistic relation of genes induced by –Glc/ + HS and + Glc/ + HS. **(D)** A picture showing the difference in gene expression extent in common genes affected both by –Glc/ + HS and + Glc/ + HS. Genes induced by –Glc/ + HS affected (loss/increase in extent) by 1.5-fold in the presence of Glc were taken into consideration. **(E)** Gene ontology enrichment of genes exclusively induced by –Glc/ + HS and + Glc/ + HS, respectively. The top 20 GO categories were plotted. Data shown are an average of three biological replicates. Venn diagrams were created using Venny v2.1; heat maps were created using Mev. Panther 15.0 tool was used to analyse GO-fold enrichment using Bonferroni correction and Fisher’s exact test type.

Out of 2,311 differentially regulated HS (−Glc/ + HS) genes, 1,124 (49%) genes were also found to be up and downregulated by Glc and HS (+ Glc/ + HS; Figure 2A and Supplementary Table 1A). Out of these 1,124 genes, 663 (59%) genes were synergistically (by both −Glc/ + HS and + Glc/ + HS) downregulated (Figures 2B,C and Supplementary Table 1A). Moreover, out of 460 (41%) –Glc/ + HS upregulated genes, the presence of Glc synergistically (upregulated by both −Glc/ + HS and + Glc/ + HS) upregulated 452 genes (Figures 2B,C and Supplementary Table 1A). Only 9 (0.8%) genes were antagonistically (−Glc/ + HS up and + Glc/ + HS down; −Glc/ + HS down and + Glc/ + HS up) regulated by both –Glc/ + HS and + Glc/ + HS. The other feature of interest was to analyse how the –Glc/ + HS-mediated induction or suppression is affected in seedlings treated with + Glc/ + HS. Among 1,124 genes, the extent of HS induction/suppression was affected (FC ≥ ± 1.5) in 259 (24%) genes in the presence of Glc (Figure 2D and Supplementary Table 1B). Glc affects the extent of HS regulation antagonistically in 174 (115 + 59; 67%) genes and agonistically in 85 (65 + 20; 33%) genes (Figure 2D). Interestingly, an exclusive set of genes was also regulated by both –Glc/ + HS (971 genes) and + Glc/ + HS (1,408 genes) categories (Supplementary Tables 1C,D). GO-upregulated categories enriched under –Glc/ + HS conditions mostly belonged to stress responses, whereas growth-related processes, such as root, embryo, seed, and fruit development, fatty acid, lipids, and other organic acids and amino acid biosynthetic processes required for organism growth, were severely downregulated (Figure 2E and Supplementary Figure 3A). In contrast, processes related to nucleotide metabolism, rRNA metabolism, and ribonucleo protein complex biogenesis required for ribosome synthesis (and ultimately leads to translation) were highly upregulated only in + Glc/ + HS (Figure 2E). Along with growth and nucleotide metabolism, we observed that Glc sufficiency (+ Glc/ + HS) exhibited high enrichment of the protein deneddylation process (Figure 2E). Protein neddylation and deneddylation are required for stress granule (SG) formation. SGs are non-membranous protein aggregates that prevent degradation of translationally halted mRNAs and store them, which can be reversibly engaged in translation once the stress is removed (Jayabalan et al., 2016; Chantarachot and Bailey-Serres, 2018). Collectively, these results indicate that plants grown under optimum energy employ different growth and stress recovery mechanisms to cope with the harmful effects of temperature stress.

### Glc *via* TOR Regulates the Transcriptome Reprogramming of HS Genes

**FIGURE 3:**
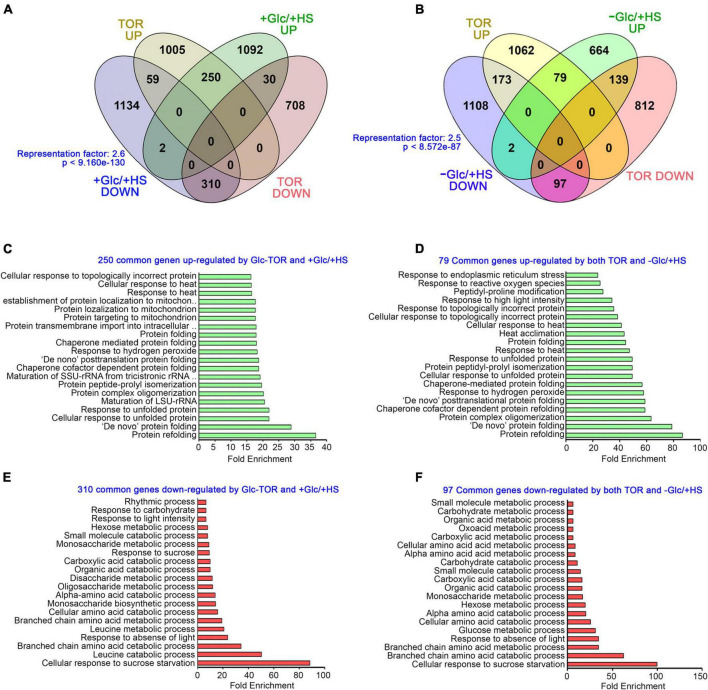
Glc *via* TOR promotes the transcriptome reprogramming of genes involved in heat stress protection. **(A)** A Venn diagram showing synergistic and antagonistic interaction of + Glc/ + HS transcriptome with publicly available Glc/TOR transcriptome of 3-day-old seedlings of WT and *tor* treated with Glc for 2 h. **(B)** A Venn diagram showing synergistic and antagonistic interaction of –Glc/ + HS transcriptome with publicly available Glc/TOR transcriptome. The statistical significance of the overlap between the two groups of genes was calculated using hypergeometric distribution provided by Nematode bioinformatics, Analysis tools, and data (http://nemates.org/). Data shown are an average of three biological replicates. **(C,E)** The GO biological process of genes commonly down and upregulated by + Glc/ + HS and public-available Glc-TOR microarray genes (Xiong et al., 2013). **(D,F)** The GO biological process enrichment of genes synergistically (up and downregulated by both Glc-TOR and –Glc/ + HS) regulated by Glc-TOR and –Glc/ + HS. The top 20 GO categories were included based on their fold enrichment. Panther 15.0 tool was used to analyse GO-fold enrichment using Bonferroni correction and Fisher’s exact test type.

The target of rapamycin, as a master regulator, senses and transduces cellular energy signals and regulates transcriptome reprogramming of myriad genes (Xiong et al., 2013). We, therefore, compared our transcriptome data with publicly available Glc/TOR transcriptome of 3 day-after-germination (DAG) seedlings of WT and *tor* treated with Glc for 2 h (Xiong et al., 2013). We found that Glc-TOR and + Glc/ + HS regulate transcriptome reprogramming largely by synergistic interaction (total 560 genes; 310 genes agonistically downregulated and 250 genes agonistically upregulated by both Glc-TOR and + Glc/ + HS) and only a small number were regulated antagonistically (total 89; 59 genes upregulated by Glc-TOR and downregulated by + Glc/ + HS and 30 genes downregulated by Glc-TOR and upregulated by + Glc/ + HS) (Figure 3A and Supplementary Table 2A). GO categories enriched under synergistic downregulation were observed to be involved in sucrose starvation and catabolic processes, such as carbohydrate, amino acids, and organic and carboxylic acid catabolism, whereas processes related to protein folding, *de novo* posttranslational protein folding, peptidyl-prolyl isomerization and protein complex oligomerization were synergistically upregulated by Glc-TOR and + Glc/ + HS (Figures 3C,E). In contrast, the –Glc/ + HS category showed major antagonism with Glc-TOR microarray (total 312 genes; 173 genes were upregulated by Glc-TOR and downregulated by –Glc/ + HS, and 139 genes were downregulated by Glc-TOR and upregulated by –Glc/ + HS) (Figure 3B and Supplementary Table 2B). In contrast, 176 genes were agonistically regulated by both Glc-TOR and –Glc/ + HS (97 genes synergistically downregulated and 79 genes were synergistically upregulated by both Glc-TOR and –Glc/ + HS) (Figure 3B and Supplementary Table 2B). GO analysis of these genes showed that 139 genes (TOR down and –Glc/ + HS up) were mostly enriched in HS acclimation and stress response (Supplementary Figure 4A). Moreover, 173 genes (up by TOR and down by –Glc/ + HS) enriched mostly belong to biosynthetic processes and DNA replication (Supplementary Figure 4B). In addition, 97 genes underlying starvation and catabolic processes were simultaneously downregulated by both TOR and –Glc/ + HS, whereas 79 core genes involved in protein folding and HS acclimation were commonly upregulated by TOR and –Glc/ + HS (Figures 3D,F). Collectively, these results suggest that Glc plays a major role in regulating HS transcriptome in a TOR-dependent manner.

### Target of Rapamycin Target E2Fa Binds Directly to HSF Gene Promoters

**FIGURE 4:**
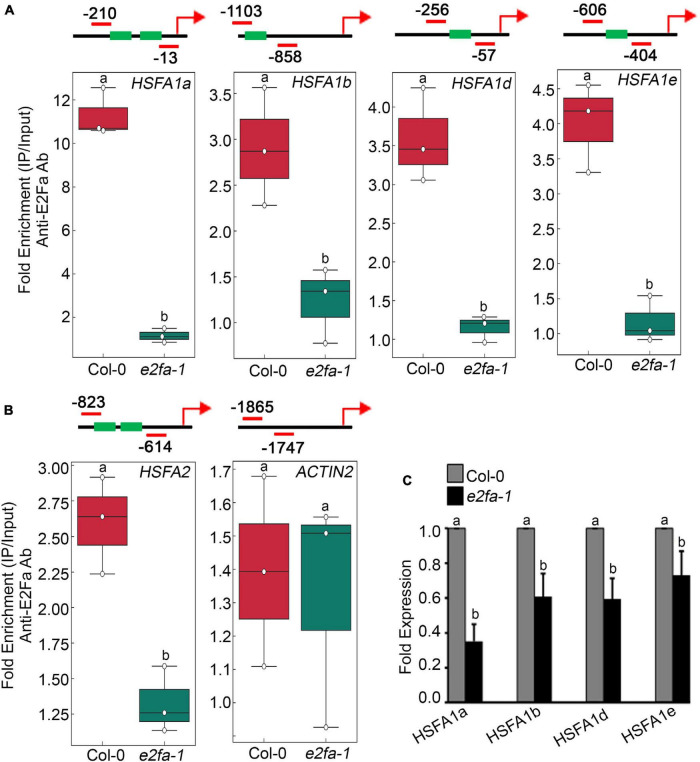
TOR phospho-substrate E2Fa binds directly to the promoters of HSF genes. **(A)** ChIP assay of *HSFA1a, HSFA1b, HSFA1d*, and *HSFA1e* promoters. Amplicon position relative to ATG of *HSFA1a*: (–210 to –13), *HSFA1b*: (–1,103 to –858), *HSFA1d*: (–256 to –57), and *HSFA1e*: (–606 to –404). **(B)** ChIP assay of *HSFA2* promoter. Amplicon position relative to ATG of *HSFA2*: (–823 to –614). In panels **(A,B)**, In the ChIP assay, *e2fa-1* mutant served as a negative background control. We also used ACT2, which does not possess any E2Fa-binding sites in its promoter region and, therefore, served as a negative target gene. Seven-day-old 0.5X MS-grown Arabidopsis Col-0 and *e2fa-1* seedlings were used for the ChIP assays. Fold enrichment of promoter fragments was calculated by comparing samples treated without or with Anti-E2Fa-specific serum. CT values without and with anti-E2Fa serum were normalized by input control. The green boxes represent putative E2F-binding elements in the HSF promoters. ChIP-qPCR analysis was performed on three technical replicates (*n* = 3) from a single representative experiment. The experiment was independently repeated two times (biological replicates; *n* = 2). Different letters denote statistical differences at *p* < 0.05 as assessed by one-way ANOVA and Tukey’s HSD *post hoc* test. **(C)** RT-qPCR showing expression of *HSFA1* genes in Col-0 and *e2fa-1* mutant. Five-day-old Arabidopsis Col-0 and *e2fa-1* seedlings were used. Experiments were independently repeated two times (biological replicates; *n* = 2). Bar plots represent mean values, and error bars denote SD. Different letters denote statistical differences at *p* < 0.05 as assessed by one-way ANOVA and Tukey’s HSD *post hoc* test.

E2Fa transcription factor binds directly to the *HLP1* promoter to activate HS gene expression (Sharma et al., 2019). Also, genes that have been shown to play a role in stress and energy signalling exhibited E2Fa binding sites in their promoters (Ramirez-Parra et al., 2003; Vandepoele et al., 2005; Xiong et al., 2013). E2Fa is a direct target of TOR that undergoes phosphorylation *via* TOR upon sugar/energy sufficiency (Xiong et al., 2013). E2Fa-binding motifs are also present in gene promoters of HSFA1 transcription factors (Ramirez-Parra et al., 2003; Naouar et al., 2009; Xiong et al., 2013). To investigate whether E2Fa directly regulates heat shock transcription factors by binding to their promoters, we performed *in vivo* ChIP assay of seven-day-old 0.5X MS-grown Arabidopsis Col-0 and *e2fa-1* seedlings using an E2Fa-specific antibody. We observed significantly higher enrichment of E2Fa on the promoters of all four *HSFA1s* in Col-0 seedlings (*HFSA1a*, *HSFA1b*, *HSFA1d*, and *HSFA1e*; Figure 4A and Supplementary Figures 5A–D). Since *HSFA2* is highly induced upon HS and is a major regulator of thermotolerance extension (Charng et al., 2006), we also looked at the E2F-binding elements in the promoter of *HSFA2*. Unexpectedly, we did not find any exact E2F-binding sites; however, we observed two E2F-like elements (TTTTTCG and TTTGGGG) in the promoter of HSFA2, which encouraged us to check whether E2Fa could also regulate HSFA2 expression through direct binding to its promoter region. In addition to conventional E2F-binding (TTTCCCGCC) elements, E2F can bind on similar E2F-like motifs in order to activate the target gene promoters (Chabouté et al., 2000; Kosugi and Ohashi, 2002). Indeed, we found occupancy of E2Fa on the *HSFA2* promoter (Figure 4B and Supplementary Figure 5E). We also used ACT2, which does not possess any E2Fa-binding sites in its promoter region and, therefore, served as a negative target gene (Figure 4B and Supplementary Figure 5F). Furthermore, transcript levels of all four *HSFA1* and *HSFA2* genes were significantly reduced in *e2fa-1* (Figure 4C; Sharma et al., 2019). These findings indicate that E2Fa transcriptionally regulates *HSFA1s* and *HSFA2* genes by directly binding to their promoters, suggesting crosstalk between TOR-E2Fa and HSFA1/HSFA2-mediated HS signalling.

### Glucose Promotes the Acetylation of Heat Stress Genes

**FIGURE 5:**
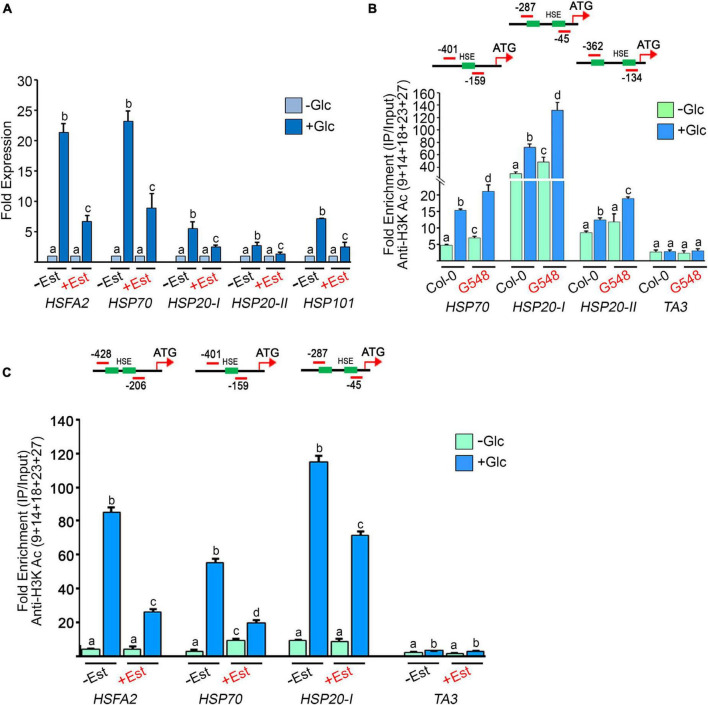
Glucose through TOR drives HS gene expression by incorporating histone H3 acetylation **(A)** RT-qPCR expression of HS genes in Arabidopsis *tor* estradiol-inducible RNAi line (*tor-es1*) under Glc lacking/sufficiency. RT-qPCR analysis was performed on three independent biological replicates (*n* = 3). Bar plots represent mean values, and error bars denote SE. Different letters denote statistical differences at *p* < 0.05 as assessed by one-way ANOVA and Tukey’s HSD *post hoc* test. **(B)** ChIP-qPCR showing enrichment of histone H3K acetylation at the promoters of HS loci under Glc lacking/sufficiency in Col-0 and TOR overexpression line G548. Seven-day-old Arabidopsis Col-0 and G548 seedlings were starved for 24 h under a sugar-deficient medium. Following starvation, seedlings were subjected to 3 h of Glc treatment (167 mM) and crosslinked with 1% formaldehyde. The data shown are representative of one biological replicate. The experiment was repeated two times with similar results. **(C)** ChIP-qPCR showing enrichment of histone H3K acetylation at the promoters of HS loci under Glc lacking/sufficiency in *tor-es1*. In panels **(A,C)**, 5-day-old *tor-es1* seedlings were transferred to 0.5X MS medium, containing 20-μM β-estradiol for 4 days. Following estradiol treatment, seedlings were starved for 24 h under the sugar-deficient medium. Following starvation, seedlings were subjected to 3 h of Glc treatment (167 mM). For mock treatment, seedlings were transferred to an equal volume of DMSO (as used for estradiol), containing MS medium. Promoter fragments containing *cis-*acting HSEs were immuno-precipitated using an anti-H3K Ac (9 + 14 + 18 + 23 + 27) antibody. The amount of immuno-precipitated promoter DNA was calculated by comparing samples treated without or with an anti-Histone H3K (9 + 14 + 18 + 23 + 27) acetyl antibody. Ct values without and with antibody samples were normalized by input control. *TA3* is a highly heterochromatinised DNA and was used as a negative control in the ChIP assays. The green boxes represent putative heat shock elements (HSEs) in the HS gene promoters. ChIP-qPCR analysis was performed on three technical replicates (*n* = 3) from a single representative experiment. The experiment was independently repeated two times (biological replicates; *n* = 2). Different letters between samples (Col-0 vs. G548 and –Est vs + Est) in each gene denote statistical differences at *p* < 0.05 as assessed by one-way ANOVA and Tukey’s HSD *post hoc* test.

According to previous microarray reports, Glc controls the transcription of a large number of HS genes (Mishra et al., 2009; Xiong et al., 2013). We confirmed these microarray results using RT-qPCR of Arabidopsis Col-0 seedlings treated with Glc. We assessed the expression of core HS-signalling genes. Glc highly induced the expression of core HS genes in comparison to plants without Glc (Figure 5A). Next, we examined HS gene expression in the TOR estradiol-inducible RNAi line (*tor-es1*) under Glc sufficiency. Arabidopsis *tor-es1* showed more than 50% reduction in HS gene expression under Glc + estradiol treatment than mock (DMSO) (Figure 5A). These results suggest that Glc regulates HS gene transcription in a TOR-dependent manner. As Glc regulates the transcripts of the HS genes through the TOR, it was interesting to investigate the mechanism of this regulation. To this end, we assessed the promoters of HS genes. Histone and chromatin modifications are important epigenetic regulators that control gene expression underlying developmental and stress responses (Chinnusamy and Zhu, 2009; Weng et al., 2014; Lee et al., 2017). The role of histone acetylation and methylation (specifically H3K4me2 and H3K4me3) in transcriptional activation underlying stress responses has already been proposed (Saleh et al., 2008; Hu et al., 2015, 2019; Ueda and Seki, 2020). We, therefore, tested the histone acetylation and methylation status at the promoters of HS genes in plants treated with and without Glc (± Glc) using a ChIP assay. The presence of Glc showed more accumulation of H3K acetylation and H3K4me2 marks at the promoters of HS genes encompassing HSEs than plants treated without Glc (Figures 5B,C and Supplementary Figure 5). We next asked whether TOR is required for Glc-induced epigenetic changes at HS gene promoters. To this end, we checked the histone epigenetic marks on the promoters of HS genes in TOR OE and *tor-es1* lines. We observed that seedlings overexpressing TOR showed increased accumulation, whereas *tor-es1* showed reduced accumulation of histone epigenetic marks under Glc sufficiency (Figure 5B and Supplementary Figures 6A,B, 7). Collectively, these results suggest that Glc regulates the expression of HS genes by epigenetically modifying their promoters. Furthermore, TOR is required for Glc-induced epigenetic changes in thermotolerance-associated genes.

### Target of Rapamycin Coordinates With HAC1 to Regulate Thermotolerance

**FIGURE 6:**
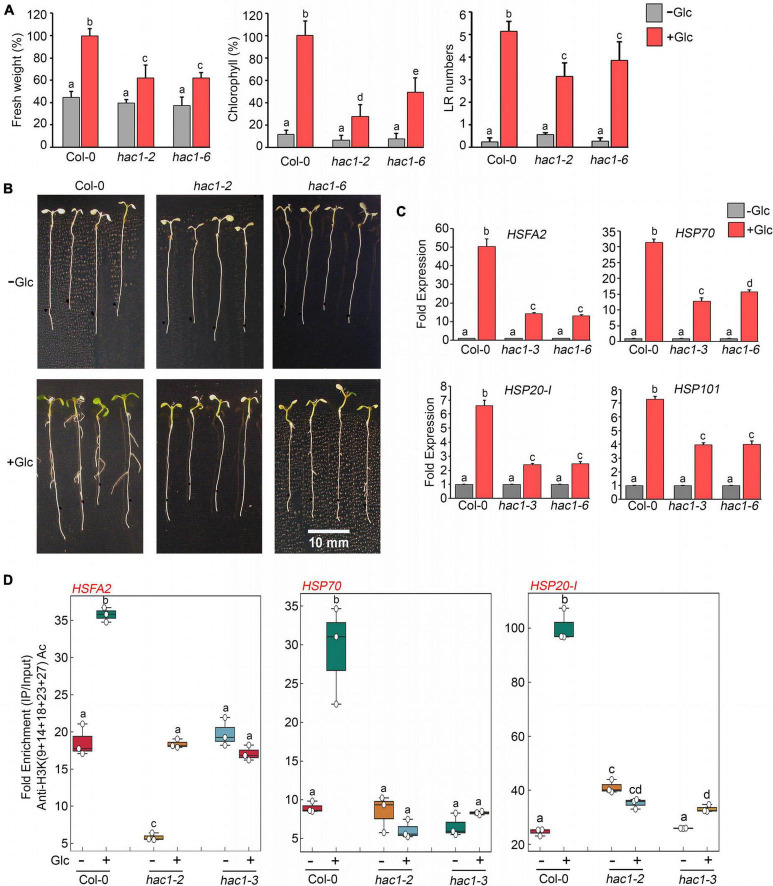
Glc-TOR-mediated HS response is governed through HAC1. **(A)** Percentage measurement of fresh weight, Chl content, and LR numbers of Arabidopsis Col-0 and *hac1* mutants after HS. **(B)** HS phenotype of Arabidopsis Col-0 and *hac1* seedlings primed with Glc (167 mM). Five-day-old 0.5X MS-grown Arabidopsis seedlings treated without or with Glc and subjected to HS. HS was applied as 1 h_37°C, 2 h_22°C, and 2.5 h_45°C, followed by recovery at 3–4 days_22°C. Physiological analysis was performed on four (Fresh weight and Chlorophyll) and three (Lateral root number) independent biological replicates (*n* = 3). Bar plots represent mean values, and error bars denote SE. Different letters denote statistical differences at *p* < 0.05 as assessed by one-way ANOVA and Tukey’s HSD *post hoc* test. **(C)** RT-qPCR expression of HS genes in Col-0 and *hac1* mutants in response to Glc (167 mM). RT-qPCR analysis was performed on three independent biological replicates (*n* = 3). Bar plots represent mean values, and error bars denote SE. Different letters denote statistical differences at *p* < 0.05 as assessed by one-way ANOVA and Tukey’s HSD *post hoc* test. **(D)** ChIP-qPCR showing enrichment of histone H3K Ac (9 + 14 + 18 + 23 + 27) at the promoters of HS gene-encompassing HSEs in Col-0 and *hac1* mutants. The amount of Immuno-precipitated promoter DNA was calculated by comparing samples treated without or with an anti-Histone H3K (9 + 14 + 18 + 23 + 27) acetyl antibody. Ct values without and with antibody samples were normalized by input control. In panels **(C,D)**, 7-day-old 0.5X MS-grown Col-0 and *hac1* seedlings were subjected to 24-h Glc starvation in MS medium without Glc and then supplied with 3-h Glc (167 mM) treatment. ChIP-qPCR analysis was performed on three technical replicates (*n* = 3) from a single representative experiment. The experiment was independently repeated two times (biological replicates; *n* = 2). Different letters denote statistical differences at *p* < 0.05 as assessed by one-way ANOVA and Tukey’s HSD *post hoc* test.

**FIGURE 7:**
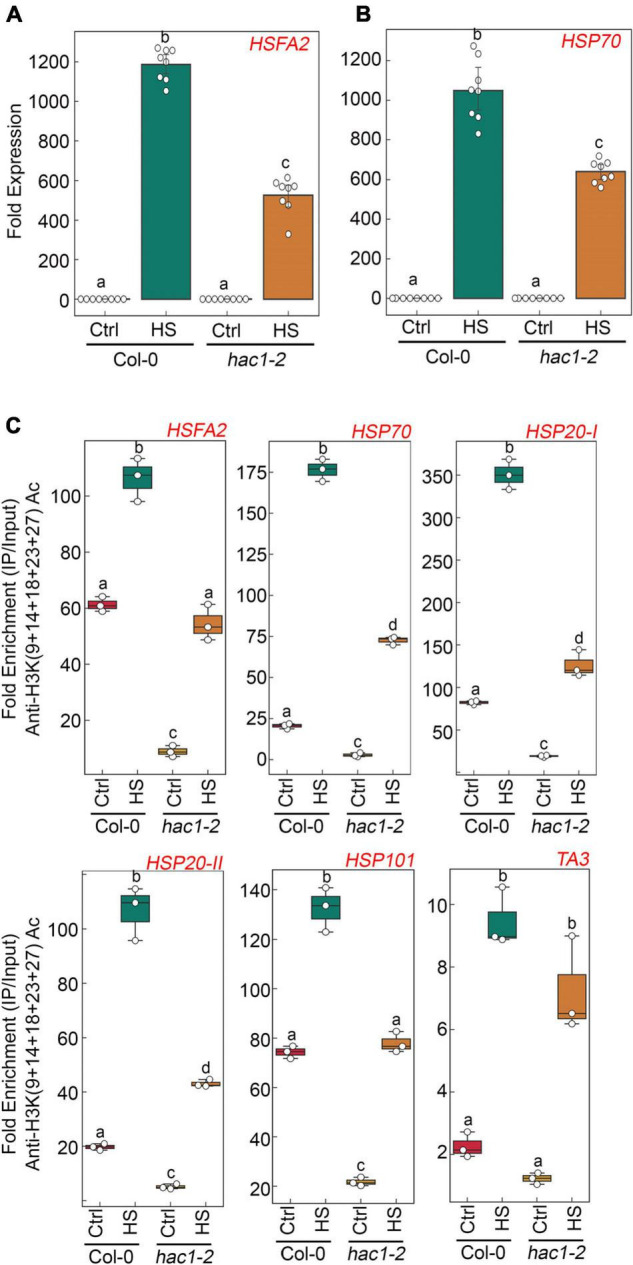
*hac1* mutant demonstrates less accumulation of H3K acetylation, leading to perturbation in HS-induced gene expression. **(A,B)** RT-qPCR expression of HS genes in Col-0 and *hac1* mutants in response to HS. Seven-day-old 0.5X MS-grown Col-0 and *hac1-2* seedlings were subjected to 3 h of HS treatment at 37°C. RT-qPCR analysis was performed on two independent biological replicates, each containing three technical replicates (*n* = 2). Different letters denote statistical differences at *p* < 0.05 as assessed by one-way ANOVA and Tukey’s HSD *post hoc* test. **(C)** ChIP-qPCR showing enrichment of histone H3K Ac (9 + 14 + 18 + 23 + 27) at the promoters of HS gene-encompassing HSEs in Col-0 and *hac1-2*. The amount of immuno-precipitated promoter DNA was calculated by comparing samples treated without or with an anti-Histone H3K (9 + 14 + 18 + 23 + 27) acetyl antibody. Ct values without and with antibody samples were normalized by input control. **(C)** Seven-day-old 0.5X MS-grown Col-0 and *hac1-2* seedlings were subjected to 3 h of HS treatment at 37°C. ChIP-qPCR analysis was performed on three technical replicates (*n* = 3) from a single representative experiment. The experiment was independently repeated two times (biological replicates; *n* = 2). Different letters denote statistical differences at *p* < 0.05 as assessed by one-way ANOVA and Tukey’s HSD *post hoc* test.

Since Glc and TOR regulate HS genes expression by modulating epigenetic status, it was important to assess how Glc/TOR controls these responses. In mammals, TOR through phosphorylation activates a CREB-binding protein/p300-type histone acetyltransferase (Wan et al., 2017). In Arabidopsis, the orthologue of p300 histone acetyltransferase HAC1 undergoes phosphorylation in response to sucrose (Van Leene et al., 2019). Moreover, HAC1, in combination with WRKY18/53, is recruited at the promoters of sugar-responsive genes and regulates their acetylation under sugar sufficiency (Chen et al., 2019). To examine whether TOR-mediated epigenetic changes at HS gene promoters are governed through HAC1, we first assessed the expression and co-expression of Arabidopsis HAC1 with TOR and RAPTOR1. Plants gauge and adapt to a diurnal light-dark cycle through transcriptional networks, which facilitate the response of plants to the external environment (Ferrari et al., 2019). We, therefore, assessed the comparative expression of *TOR* and *HAC1* in diurnal light-dark cycle transcriptome data obtained from public resources and identified that TOR expression overlaps with *HAC1* expression throughout the diurnal cycle (Ferrari et al., 2019; Supplementary Figure 8A). Genevestigator^1^ and string^2^ data also suggest that *HAC1* shares huge similarities with *TOR* and *RAPTOR1* in expression and prediction of protein-protein interactions (Szklarczyk et al., 2019) (Supplementary Figures 8B–D, 9). Therefore, to assess whether HAC1 regulates thermotolerance response, we examined the phenotype of Arabidopsis plants defective in *HAC1* in response to HS. Seven-day-old Arabidopsis Col-0 and *hac1* mutants (*hac1-2* and *hac1-6*) were subjected to Glc-free and Glc-containing MS media for 24 h, followed by short-term-acquired thermotolerance (SAT) HS treatment. As expected, Col-0 and *hac1* mutants treated without Glc could not survive the lethal HS. However, Col-0 plants supplemented with Glc showed improved thermotolerance [showed higher fresh weight, increased chlorophyll (Chl), and higher lateral root numbers] (Figures 6A,B). Interestingly, *hac1* mutants showed reduced fresh weight and Chl content and fewer lateral roots after HS (Figures 6A,B). Next, we assessed the expression levels of HS genes underlying thermotolerance response in Col-0 and *hac1* mutants using RT-qPCR. Col-0 plants subjected to Glc and HS showed increased expression of these HS genes (Figures 6C, 7A,B and Supplementary Figure 10). In contrast, alleles of *hac1* (*hac1-2*, *hac1-3*, and *hac1-6*) revealed greater perturbation in HS gene expression, following sugar and HS treatment (Figures 6C, 7A,B and Supplementary Figure 10). The next important question was to assess the epigenetic status of HS gene promoters upon sugar and HS treatment in Col-0 and *hac1* mutants. We, therefore, performed ChIP assay to examine the acetylation levels of HS gene promoters using an anti-H3K (9 + 14 + 18 + 23 + 27) acetylation antibody coupled with qPCR. Intriguingly, Arabidopsis Col-0 plants showed increased accumulation of H3K acetylation at HS gene promoters upon sugar and HS treatment, whereas plants having a mutation in *HAC1* showed reduced acetylation at these gene promoters (Figures 6D, 7C and Supplementary Figures 11, 12). Collectively, these findings suggest that Glc-TOR signalling coordinates with the epigenetic machinery to regulate thermotolerance.

**FIGURE 8:**
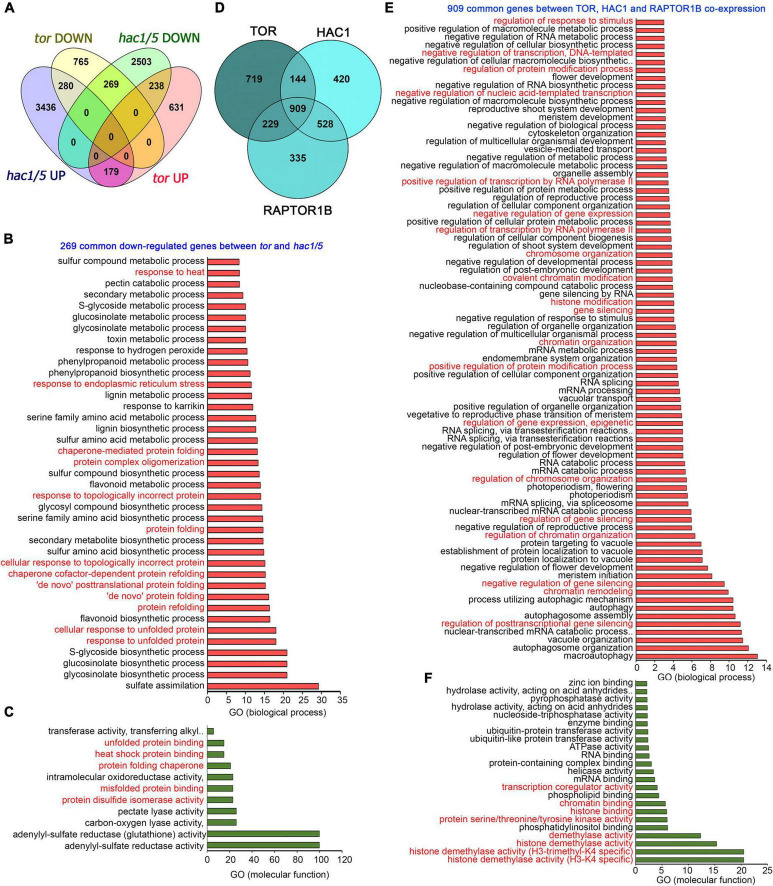
Transcriptome comparison of TOR microarray with differentially expressed genes in *hac1*. **(A)** A Venn diagram showing overlap of genes differentially expressed in *tor* and *hac1/5* mutants. Transcriptome data of TOR and HAC1 were obtained from public resources (Xiong et al., 2013; Jin et al., 2018). Genes in *hac1/5* mutants were extracted based on their false discovery rate value less than.05. **(B,C)** The GO biological process and molecular function of 269 genes commonly downregulated by *tor* and *hac1/5*. **(D)** A Venn diagram showing overlap of co-expression genes between TOR, HAC1, and RAPTOR1B. Co-expression genes were extracted from the ATTED-II database, and a total of 2,000 genes were used for co-expression genes overlap. **(E)** The GO biological process of 909 commonly co-expressed genes between TOR, HAC1, and RAPTOR1B. **(F)** GO molecular function of 909 commonly co-expressed genes between TOR, HAC1, and RAPTOR1B. Panther 15.0 tool was used to analyse GO-fold enrichment using Bonferroni correction and Fisher’s exact test type.

In addition, a global transcriptome comparison of *tor* differentially expressed genes (DEGs) with *hac1/5* obtained from public resources (Jin et al., 2018) identified 269 commonly downregulated genes (Figure 8A). GO biological process categorization of these 269 commonly regulated genes displayed high enrichment of protein folding, refolding, response to unfolded or topologically incorrect proteins, protein complex oligomerization, and response to ER and heat stress (Figure 8B). GO molecular function also indicated the high enrichment of protein disulphide isomerase activity, heat shock protein binding, and misfolded and unfolded protein binding (Figure 8C). Moreover, TOR and RAPTOR1 exhibit a huge overlap of co-expressed genes with HAC1 having high enrichment of processes involved in chromatin remodelling, histone modification, and gene transcriptional/posttranscriptional regulation (Figures 8D–F). These *in silico* analyses suggest that TOR, together with HAC1, may regulate the expression of heat stress-associated genes, leading to thermotolerance.

## Discussion

During their life cycle, plants face adverse growth conditions. An environmental factor that significantly affects plant growth and reproduction is an increase in temperature above optimum. Being sessile, plants cannot move to more favourable locations and, therefore, must adapt to these stressful temperatures through an inherent potential called basal or acquired thermotolerance. A well-studied mechanism to provide thermoprotection is through the induction of HSPs that protect the cellular proteome against heat-induced damage. Previously, numerous researchers have identified the involvement of TOR in stress resilience and adaptation (Mahfouz et al., 2006; Deprost et al., 2007; Wang et al., 2016, 2018; Bakshi et al., 2017; Dong et al., 2017, 2018; Fu et al., 2020). Glc signalling controls the transcription of many heat shock protein (HSP) genes, including *HSFA2* and *HSP70* (Sharma et al., 2019). In addition, a global transcriptome study of Arabidopsis plants under Glc sufficiency or plants underexpressing TOR (*tor35-7 RNAi*) showed that Glc-TOR signalling governs transcriptional reprogramming of HS genes (Mishra et al., 2009; Xiong et al., 2013; Gupta et al., 2015b). However, the underlying mechanism of how TOR controls thermotolerance response is not well understood. Through a comprehensive chemical, genetic, transcriptome, and epigenetic analysis, our results presented here provided the pivotal role of Arabidopsis Glc-TOR signalling in heat stress adaptation.

Previously, it has been reported that Glc signalling activates root apex, whereas both Glc and light are required to activate the shoot apex (Li et al., 2017). Glc-TOR signalling is vital for the activation of *pCYCB1:GUS*, a reporter of active cell proliferation, following heat stress recovery in SAM (Sharma et al., 2019). In agreement with the previous observations, plants grown under HL without Glc showed less growth and stress recovery than plants treated under HL with Glc suggesting an essential role of Glc in stress adaptation and recovery. We hypothesise that reactivation of proliferation competent cells in the SAM might have high energy demands following heat stress. Also, light alone was not sufficient to reactivate proliferation competent cells to support growth from the shoot apex. Light may also require other signals, such as Glc and auxin, to activate TOR in the shoot apex (Andriankaja et al., 2012; Li et al., 2017; Sharma et al., 2019).

Transcriptome study of Arabidopsis seedlings under Glc lacking/sufficiency demonstrated that Glc largely affects the expression of HS-related genes. At the whole genome level, 1,124 genes were co-regulated by −Glc/ + HS and + Glc/ + HS. As expected, several metabolic processes related to primary and secondary metabolisms, microtubules disassembly, sulphur compound biosynthetic processes, and biotic stimulus were severely downregulated, whereas genes involved in HS protection, such as HS-responsive molecular chaperones, *de novo* protein folding, HS acclimation, and *de novo* posttranslational protein folding were upregulated (Supplementary Figures 2A,B). Interestingly, apart from common genes regulated by −Glc/ + HS and + Glc/ + HS, the presence of Glc exclusively regulates 1,408 genes. GO enrichment of these genes showed high enrichment of the protein deneddylation process (Figure 2E). Protein neddylation and deneddylation upon stress stimuli and stress recovery are required for the assembly and disassembly of stress granules (SG), respectively (Jayabalan et al., 2016). Like ubiquitylation, neddylation is a posttranslational modification, which requires small ubiquitin-like protein NEDD8. Under stress, organisms show inhibition in protein synthesis, block translation initiation, and trigger polysome disassembly and sequestration of translation-stalled mRNA into cytoplasmic non-membranous aggregates called SG for storage or in processing bodies (PBs) for decay (Chantarachot and Bailey-Serres, 2018). SGs prevent the degradation of mRNA and store them, which can reversibly be engaged into the translation once the stress has been removed. Cells employ these mechanisms to repress the translation of housekeeping genes and preserve stress-protective genes mRNA to ensure cell survival. Recently, Merret et al. (2017) have shown that mutants of *HSP101* showed perturbation in translational recovery and SG disassembly following HS (Merret et al., 2017). The mRNA-encoding ribosomal proteins (RPs) were stored into SGs, and subsequent release of this mRNA in re-engaging translation process was dependent on HSP101 (Merret et al., 2017). In agreement with the previous reports, our data strongly suggest that Glc signalling may drive regulation of SG assembly and disassembly through the expression of genes underlying neddylation and deneddylation, which are crucial for stress recovery. In addition, Glc through TOR promotes the transcriptional regulation of *HSP101*, which is crucial for SGs disassembly and, hence, provides an adaptive strategy of plant survival under deleterious HS. Transcriptome reprogramming of HS genes under Glc sufficiency exhibited major synergistic interaction with TOR-regulated genes, suggesting that Glc may govern transcriptome reprogramming of HS genes mainly through the TOR pathway.

Earlier reports have shown that Glc regulates transcriptome reprogramming of a myriad set of genes *via* the E2Fa transcription factor (Xiong et al., 2013; Li et al., 2017). E2Fa regulates target genes *via* conserved *cis-*acting TTTCCCGCC or similar motifs through direct binding at their genomic sites (Vandepoele et al., 2005; Naouar et al., 2009). Using ChIP-assay, we observed that E2Fa protein occupies the promoters of *HSFA1s* and *HSFA2* genes and positively regulates their expression.

Numerous reports revealed that the dynamic nature of chromatin structure is a crucial determinant of gene expression and can be modulated by nucleosome rearrangement, histone variant, and posttranslational modification of histone tails (Berr et al., 2009; Tamada et al., 2009; Kumar and Wigge, 2010; Brzezinka et al., 2016). Depending on the position of histone residues, which are acetylated or methylated, these modifications can promote or repress transcription. There are numerous reports, which suggested the role of histone acetylation and methylation (specifically H3K4 di- and tri-methylation) in gene transcriptional activation underlying stress responses (Saleh et al., 2008; Hu et al., 2015, 2019; Ueda and Seki, 2020). Recently, Chen et al. (2019) have shown that p300/CREB HISTONE ACETYLTRANSFERASE 1 (HAC1), in association with WRKY18/53 transcription factors, regulates sugar-responsive gene expression by modulation of histone acetylation (Chen et al., 2019). Our study provides strong evidence supporting that Glc-TOR signalling regulates HS transcripts by the accumulation of histone acetylation marks at the promoters of HS loci. We observed a strong defect of *tor* mutant on Glc-promoted HS expression, demonstrating that Glc induction of HS transcriptome is majorly regulated through TOR. In mammals, TOR through phosphorylation activates a CREB-binding protein/p300-type histone acetyltransferase (Wan et al., 2017). In Arabidopsis, the orthologue of p300 histone acetyltransferase HAC1 undergoes phosphorylation in response to sucrose (Van Leene et al., 2019). Moreover, HAC1, in combination with WRKY18/53, is recruited at the promoters of sugar-responsive genes and regulates their acetylation under sugar sufficiency (Chen et al., 2019). In our study, we observed that *hac1* showed hypersensitivity to HS with a strong defect in Glc-promoted accumulation of H3K acetylation, leading to reduced expression of the HS gene. Furthermore, a global transcriptional comparison of TOR and HAC1 showed an overlap of 269 genes, with GO categories implicated in heat shock responses. Based on the co-expression data of TOR and HAC1 and reduced acetylation of HSP gene promoters, as well as reduced thermotolerance of their mutants, we speculate that TOR and HAC1 may act together in regulating the heat stress response. However, the precise molecular mechanism of how TOR and HAC1 regulate this response is unclear at this point. It is plausible that, like mammalian TOR, Arabidopsis TOR may phosphorylate HAC1 and activate it, which transcriptionally regulates the chromatin landscape of the HSP genes to confer thermotolerance. Moreover, TOR and HAC1 regulate similar pathways, for example, in mammals, mTORC1-mediated phosphorylation of p300 suppresses cellular autophagy and activates cell lipogenesis (Wan et al., 2017). HAC1 also serves several other functions; one of which is leaf senescence that is governed by TOR (Deprost et al., 2007; Hinckley et al., 2019). Autophagy is the self-digestion of its cellular components. In addition to normal growth and developmental programmes, stress responses also activate the cellular autophagy response (Fulda et al., 2010; Kroemer et al., 2010). Through negative regulation of autophagy during heat stress, TOR and HAC1 may confer increased thermotolerance during heat stress. Future work in this direction will elucidate the exact mode of action of the TOR–HAC1 interaction.

**FIGURE 9:**
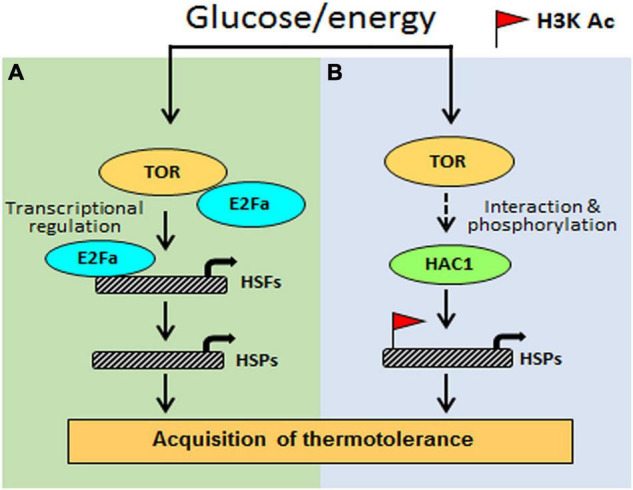
The proposed model shows how Glc-TOR signalling regulates thermotolerance. **(A)** TOR-regulated E2Fa transcription factor recruited at the promoters of *HSFA1* and *HSFA2* genes to control their expression, which, in turn, regulate HS gene expression. **(B)** Glc through TOR promotes recruitment of histone H3 acetylation marks at the promoters of HS genes to induce their transcription, leading to thermotolerance. This Glc-TOR-mediated histone acetylation is facilitated through HAC1.

Based on the investigation, we propose a testable model in Figure 9. Arabidopsis Glc-TOR signalling plays a pivotal role in providing thermotolerance by modulating the chromatin landscape of thermo-responsive loci. TOR through distinct mechanisms controls thermotolerance response. Glc-activated TOR *via* the E2Fa transcription factor governs the transcriptome reprogramming of a large set of heat shock genes. Moreover, Arabidopsis TOR works in coordination with histone acetylation machinery to modulate thermotolerance response. As the TOR signalling is also present in humans and is highly conserved across species, this finding will have larger implications and could be translated into other species. The conserved nature of TOR would interest other researchers working in similar areas. Also, this work identified the crucial function of TOR in stress adaptation and provides a mechanistic framework to confront ever challenging and fluctuating environments.

## Materials and Methods

### Plant Materials and Growth Condition

The *Arabidopsis thaliana* mutant lines estradiol-inducible *tor* RNAi (CS69829; *tor-es1*; Col-0 background), TOR OE (AT1G50030, GK-548G07- 020632; Col-0 background), and *e2fa* (AT2G36010, WiscDsLox434F1; Col-0 background) were obtained from ABRC at Ohio State University. The following lines were obtained from the published sources: *tor35-7* RNAi [Col-0 background; (Deprost et al., 2007)] and *HAC1* mutants [*hac1-2*, *hac1-3*, and *hac1-6*; Col-0 background (Heisel et al., 2013)], *proTOR:GUS* [Col-0 background (Menand et al., 2002)]. Seeds were sown on square petri plates (120 mm × 120 mm) containing 0.5X MS medium with 1% Suc (29 mM) and.8% agar (24 mM) under long-day photoperiod [16-h light and 8-h dark, 60 micro mols (μmol) m^–2^ s^–1^ light intensity, otherwise stated in the figure legend] at 22°C ± 2°C temperature.

### Chl Estimation

Total Chl (Chl a and Chl b) estimation was performed with seedlings treated with HS. Chl estimation was described by Kushwah and Laxmi (2014).

### ChIP Assays

ChIP assays were performed using the protocol described by Saleh et al. (2008) with minor modifications. For E2Fa ChIP, 7-day-old 0.5X MS-grown Col-0 and *e2fa* seedlings were used. For acetylation and H3K4me2 ChIP assay, 5-day-old *tor-es1* seedlings were transferred to 0.5X MS medium containing 20-μM β-estradiol for 4 days. Following estradiol treatment, seedlings were starved for 24 h under a Glc-deficient medium. Following starvation, seedlings were subjected to 3 h of Glc treatment (167 mM). Briefly, 1-gm tissue of each sample was cross-linked with 1% formaldehyde to fix protein-DNA complexes. The samples were crushed in liquid N2 and homogenised in nuclei isolation and nuclei lysis buffers, followed by sonication. Sonication of chromatin was done in a 4°C water sonicator (DiagenodeBioruptor Plus). Sonicated samples were first precleared with Protein A Agarose beads (Millipore #16-157) and then mixed with antibodies. Serum containing anti-E2Fa antibodies was obtained from Prof. Lieven De Veylder, VIB Department of Plant Systems Biology, Ghent University (Takahashi et al., 2008). Antibodies against H3K (9 + 14 + 18 + 23 + 27) acetylation were purchased from Abcam (Cat. No. ab47915). Antibodies against H3K4me2 (Cat. No. 07-030) were purchased from Millipore. All primers used are mentioned in Supplementary Table 3.

### Thermotolerance Assays

*Arabidopsis thaliana* seedlings grown in a standard culture room and maintained under long-day photoperiod conditions were used for thermotolerance assays. For phenotyping under low-light (LL) and high-light (HL) conditions, Arabidopsis seedlings were grown directly onto 0.5X MS medium containing without or with Glc (90 mM) for 7 days under LL and HL intensities and then transferred to HS. HS was applied as 1 h_37°C, 2 h_22°C, and 2.5 h_45°C, followed by recovery at 3–7 days_22°C under normal light (5000 LUX; NL) intensity. For thermotolerance of *hac1* mutants, 5-day-old seedlings were grown initially in MS media, containing 1% sucrose (29 mM) and.8% Agar and then acclimatised to Glc-free and Glc-containing MS media for 24 h, followed by HS treatments. HS was applied as 1 h_37°C, 2 h_22°C, and 2.5 h_45°C, followed by recovery at 3–4 days_22°C.

### Gene Expression Analysis

For quantitative real-time PCR (RT-qPCR) study in response to Glc, 5-day-old 0.5X MS-grown Arabidopsis seedlings were starved without Glc for 24 h in dark, followed by 3 h of Glc treatment in the light. For RT-qPCR study under HS, Arabidopsis seedlings were germinated and grown directly in 0.5X MS medium, containing sucrose (29 mM) for 7 days under standard 16-h-day/8-h-night conditions and subjected to HS at 37°C for 3 h. Total plant RNA was isolated using RNA easy Plant Mini Kit (Qiagen), and cDNA was prepared using 2 μg of total RNA with a high-capacity cDNA Reverse Transcription Kit (Applied Biosystems). All candidate gene primers were designed using a unique sequence within the genes, preferably 3′UTR. The normalization of genes was performed using 18S as a reference control. The fold-change for each candidate gene in different experimental conditions was determined using the quantitative ΔΔCT method. All primers used are listed in Supplementary Table 3.

### Microarray Analysis

Whole-genome microarray analysis of 5-day-old Arabidopsis Col-0 seedlings treated without or with Glc (−/ + Glc) and HS was performed. To minimise the endogenous sugar background, 5-day-old Col-0 seedlings were first starved with 0.5X MS medium without sugar for 24 h in the dark, followed by 24-h Glc (167 mM) in the light and then subjected to HS. HS treatment was applied at 37°C for 3 h and 37°C for 3 h, followed by recovery at 22°C for 3 h. Harvested samples were outsourced for transcriptome profiling. The data were analysed using the Transcriptome Analysis Console (v3.0; Affymetrix) using default parameters. The differentially expressed genes (DEGs) in all conditions were compared to −Glc with fold change (FC) value (≥1.5). One-way between-subject ANOVA (unpaired) statistical test, with *p*-value less than.05 and false discovery rate (FDR) *p*-value.1, was used to analyse the DEGs. Microarray data have been submitted to Array Express with accession No. E-MTAB-6980. Three biological replicates were used in this microarray study.

### Bioinformatics Analysis

Gene expression data for TOR, RAPTOR1, and HAC1 in *A. thaliana* across various anatomical parts, developmental stages, and stress-related experiments were extracted using the mRNA-Seq dataset “AT_mRNASeq_ARABI_GL” from Genevestigator platform (Hruz et al., 2008). The available time-series expression data (in transcripts per million TPM counts) for Arabidopsis diurnal range across the day-night cycle were downloaded from the Supplementary Dataset in Ferrari et al. (2019). Temporal expression patterns for all four genes were extracted from this dataset and compared using Normalized TPM values (rescaled between 0 and 1 *via* Min-Max scaling/normalization). Expression heat maps were generated using MeV_4_9_0.

## Data Availability Statement

The original contributions presented in the study are publicly available. This data can be found here: Microarray data have been submitted to Array Express with accession no. E-MTAB-6980.

## Author Contributions

MoS and AL conceived and designed the experiments and analysed the data. MoS performed most of the experiments. MoS and MJK performed the microarray experiment. MoS, BNS, MaS, and PA performed the Physiology and RT-qPCR experiments. MoS, SM, and GY performed the Bioinformatic analysis. MoS wrote the manuscript. MJK read the manuscript critically. AL supervised and complemented the writing. All authors contributed to the article and approved the submitted version.

## Conflict of Interest

The authors declare that the research was conducted in the absence of any commercial or financial relationships that could be construed as a potential conflict of interest.

## Publisher’s Note

All claims expressed in this article are solely those of the authors and do not necessarily represent those of their affiliated organizations, or those of the publisher, the editors and the reviewers. Any product that may be evaluated in this article, or claim that may be made by its manufacturer, is not guaranteed or endorsed by the publisher.

## Footnotes

1
https://genevestigator.com/

2
http://string-db.org/
