# Supplementary material for: Arabidopsis Target of Rapamycin Coordinates With Transcriptional and Epigenetic Machinery to Regulate Thermotolerance
Source: Front Plant Sci. 2021 Oct 28;12:741965. doi: 10.3389/fpls.2021.741965 (PMC8581614; doi:10.3389/fpls.2021.741965)
Supplement: Supplementary file 4 [file Data_Sheet_1.docx]

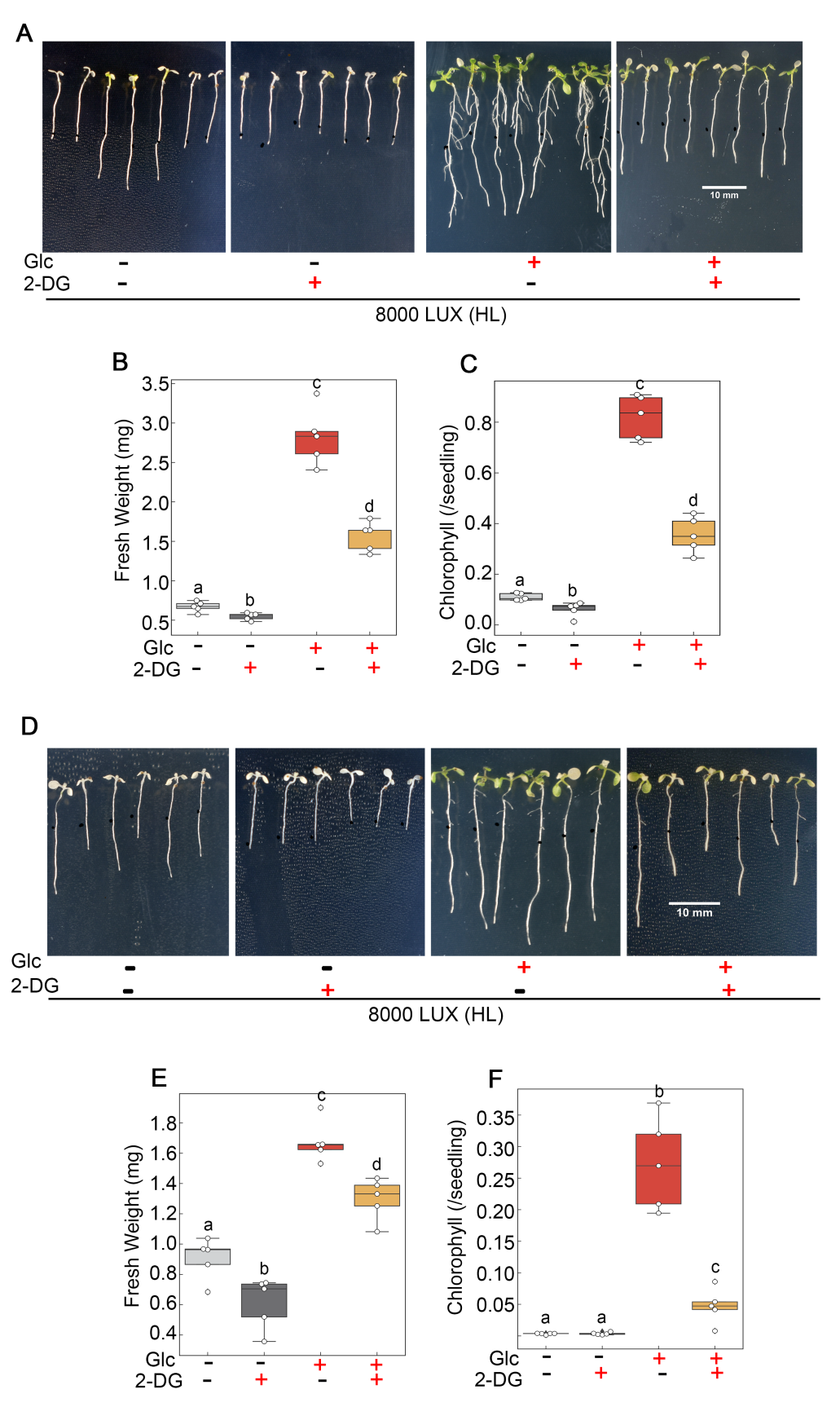


**Supplemental Figure S1. Photosynthesis generated Glc could not provide thermotolerance under high light intensity when blocked with Glc analogue 2-deoxy-glucose. A-C,** Phenotype, FW and Chl measurement of Arabidopsis Col-0 seedlings grown initially for five days on 0.5X MS medium containing 1% sucrose under high light condition and then subjected to Glc and 2-DG treatment. Arabidopsis Col-0 seedlings were grown for five days on 0.5X MS medium containing 1% sucrose and high light (8000 LUX) intensity. Seedlings were then transferred to without or with Glc (90 mM) containing MS media without or with Glc analogue 2-DG (2.5 mM) and kept for 48h under high light intensity. After 48h acclimation, seedlings were subjected to heat stress at 1h_37ºC, 2h_22ºC, 2.5h_45ºC and 3-7d_22ºC. **D-F,** Phenotype, FW and Chl measurement of Arabidopsis Col-0 seedlings grown initially for five days under Glc deficient media under high light condition and then subjected to Glc and 2-DG treatment. Col-0 seedlings were grown for five days on Glc deficient medium under high light (8000 LUX) intensity. Seedlings were then transferred to without or with Glc (90 mM) containing MS media without or with Glc analogue 2-DG (2.5 mM) and kept for 48h under high light intensity. After 48h acclimation, seedlings were subjected to heat stress at 1h_37ºC, 2h_22ºC, 2.5h_45ºC and 3-7d_22ºC. Phenotypic analysis was performed on five independent biological replicates (n=5). Different letters denote statistical differences at P<0.05 as assessed by one-way ANOVA and Tukey’s HSD posthoc test.


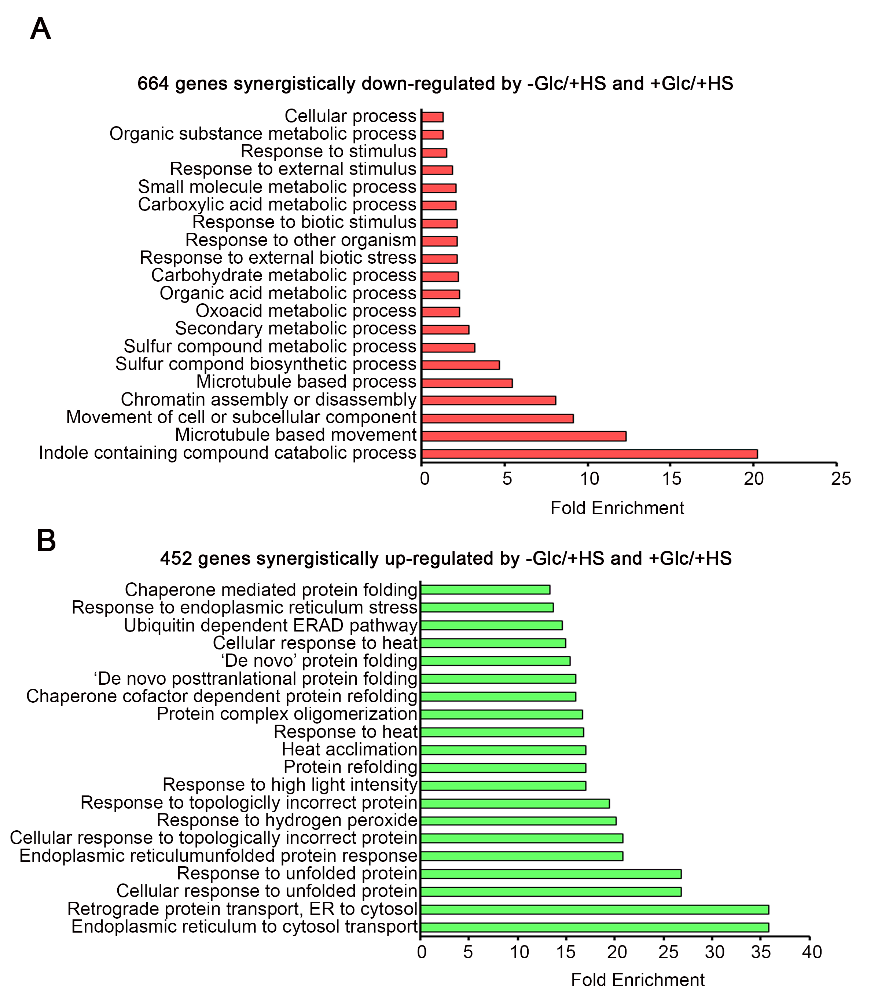


**Supplemental Figure S2. Gene ontology of common genes between –Glc/+HS and +Glc/+HS exhibits up-regulation of genes involved in protein folding. A, B** GO term enrichment of biological process synergistically down-and up-regulated by both –Glc/+HS and +Glc/+HS. The top twenty GO categories were included based on their fold enrichment. Panther 15.0 tool was used to analyse GO fold enrichment using Bonferroni correction and Fisher’s exact test type.


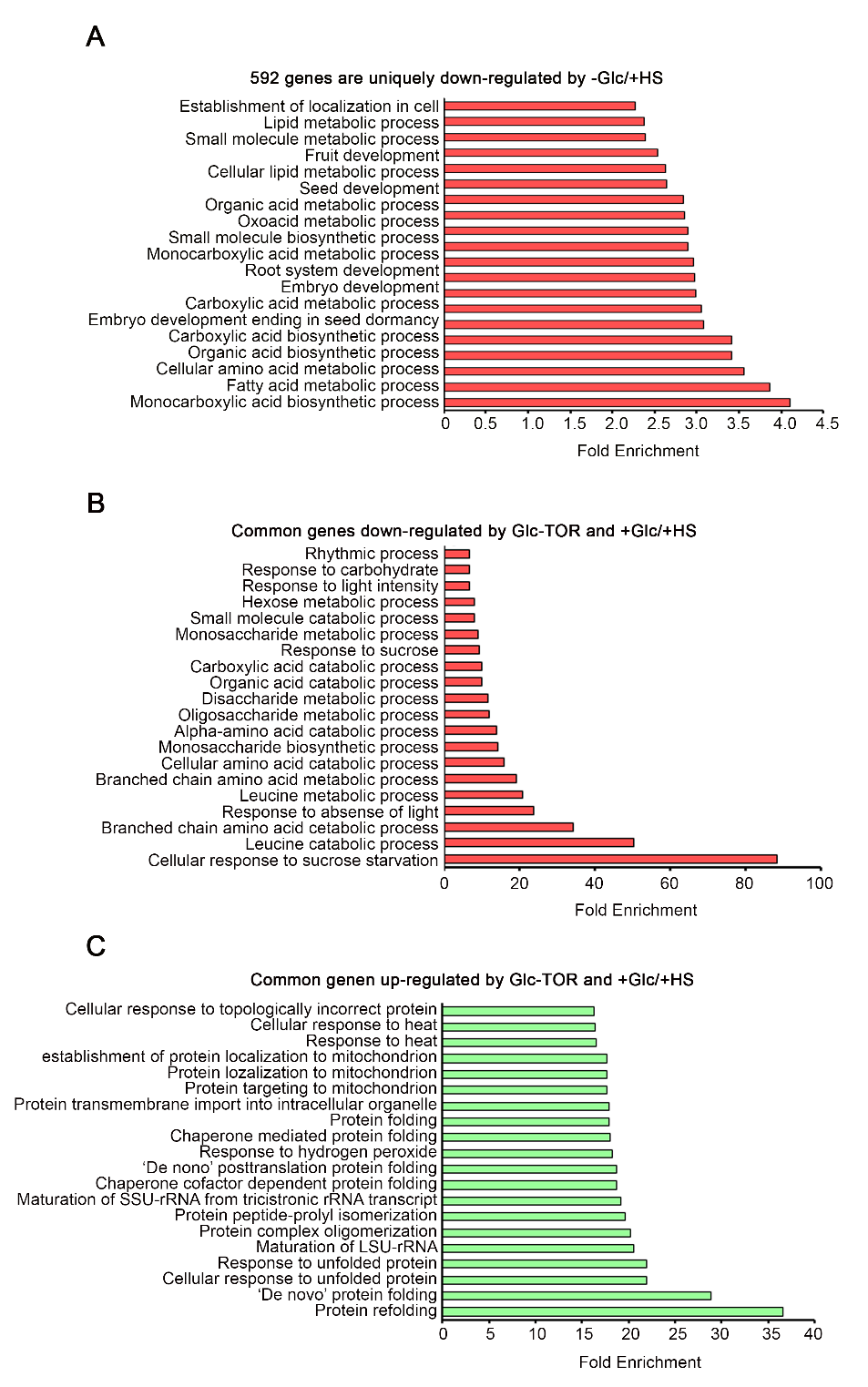


**Supplemental Figure S3. Glc regulates HS transcriptome largely through the TOR pathway. A,** GO biological process of genes exclusively down-regulated by –Glc/+HS. The top twenty GO categories were included based on their fold enrichment. Panther 15.0 tool was used to analyse GO fold enrichment using Bonferroni correction and Fisher’s exact test type.


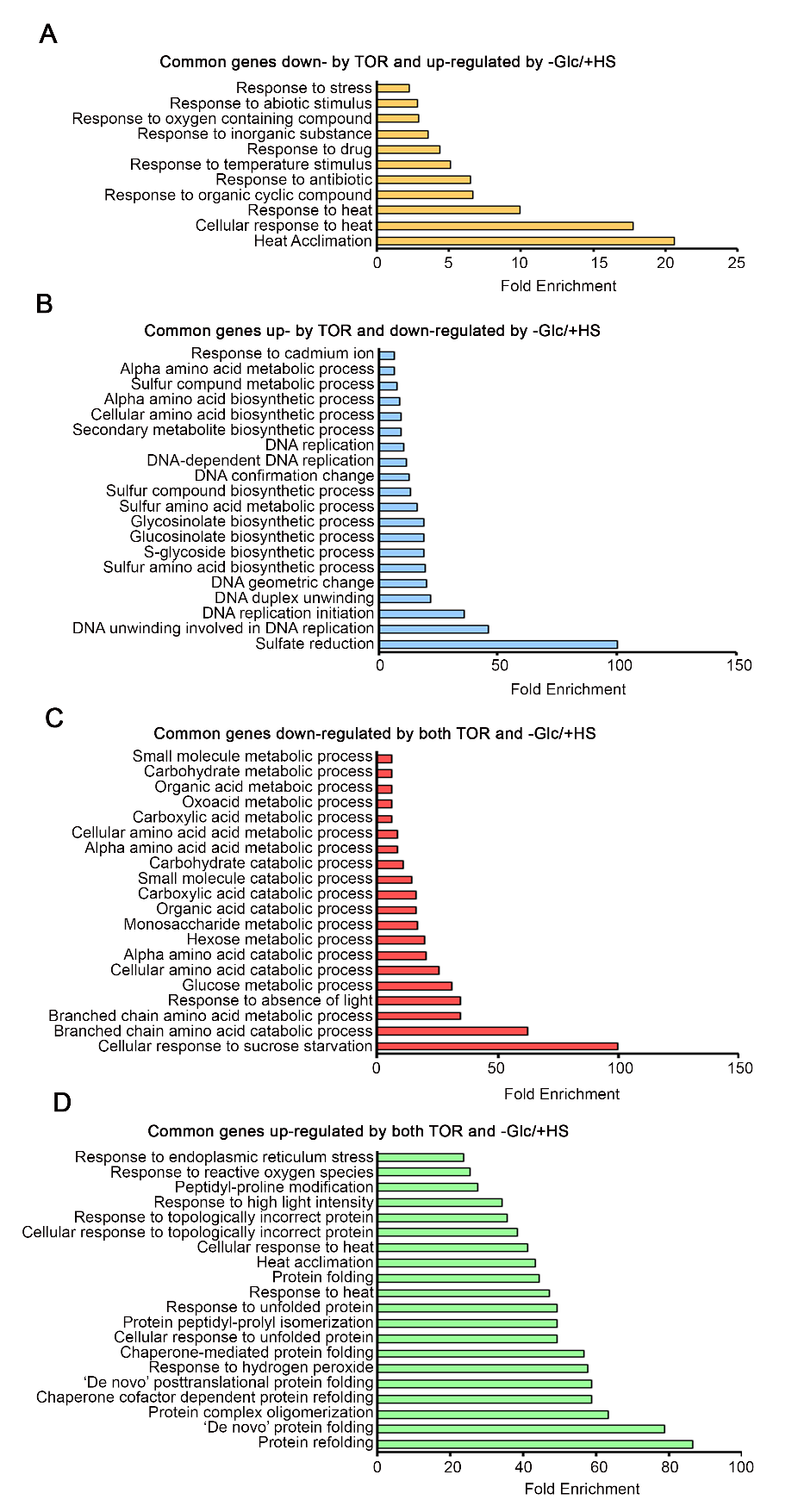


**Supplemental Figure S4. HS transcriptome induced in absence of Glc exhibits large antagonistic interaction with Glc-TOR target genes. A, B,** GO biological process of genes antagonistically (down- by Glc-TOR and up-regulated by –Glc/+HS; up- by Glc-TOR and down-regulated by –Glc/+HS) regulated by Glc-TOR and –Glc/+HS. The top twenty GO categories were included based on their fold enrichment. Panther 15.0 tool was used to analyse GO fold enrichment using Bonferroni correction and Fisher’s exact test type.


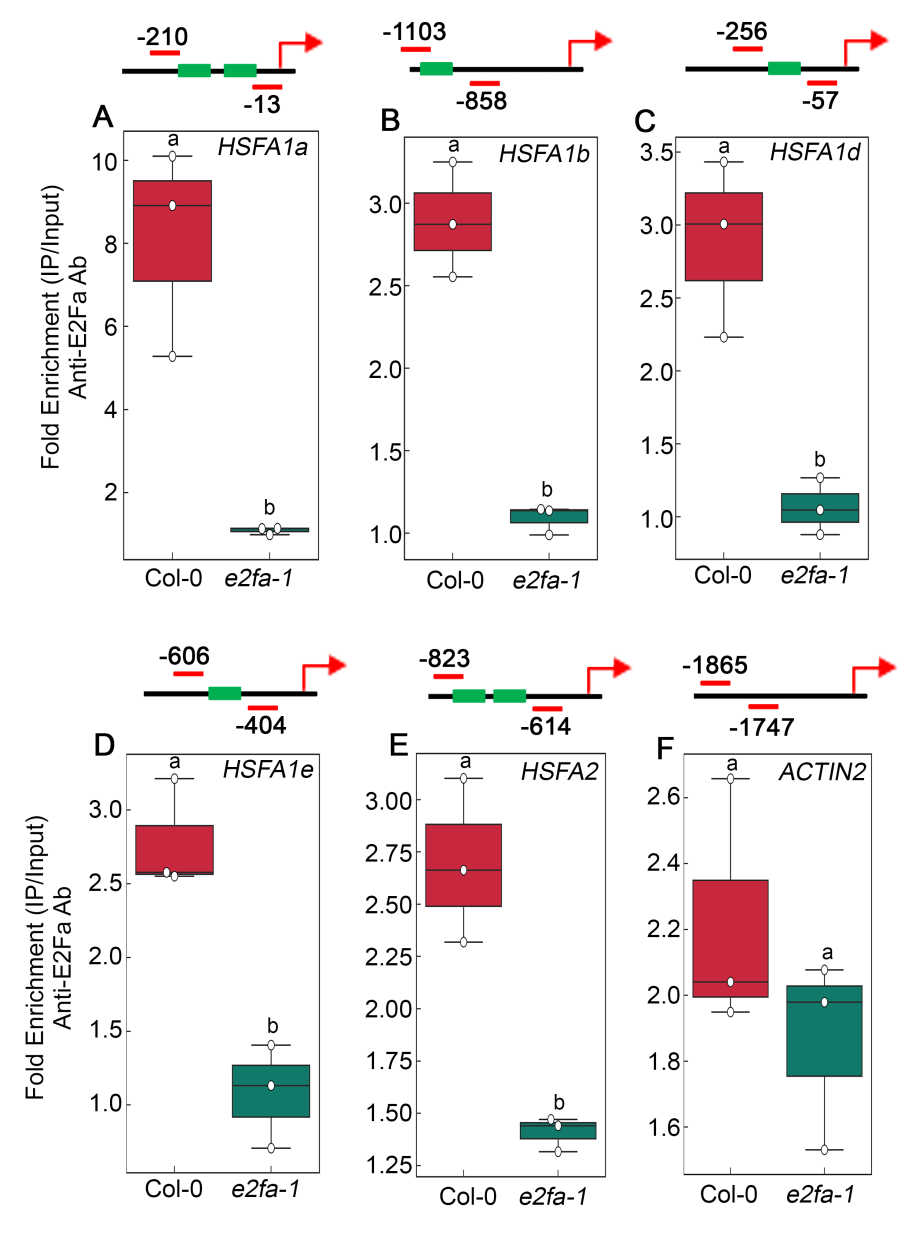


**Supplemental Figure S5. Second biological replicates showing occupancy of E2Fa on the promoters of *HSFA1* and *HSFA2* genes. A-D,** ChIP assay of *HSFA1a, HSFA1b, HSFA1d* and *HSFA1e* promoters. **E,** ChIP assay of *HSFA2* promoter. In the ChIP assay, *e2fa-1* mutant served as a negative background control. We also used *ACT2* which does not possess any E2Fa binding sites in its promoter region and therefore, served as a negative target gene. Seven-day-old 0.5X MS grown Arabidopsis Col-0 and *e2fa-1* seedlings were used for the ChIP assays. ChIP-qPCR analysis was performed on three technical replicates (n=3) from a single representative experiment. Experiment was independently repeated twice (biological replicates; n=2). Different letters denote statistical differences at P<0.05 as assessed by one-way ANOVA and Tukey’s HSD posthoc test.


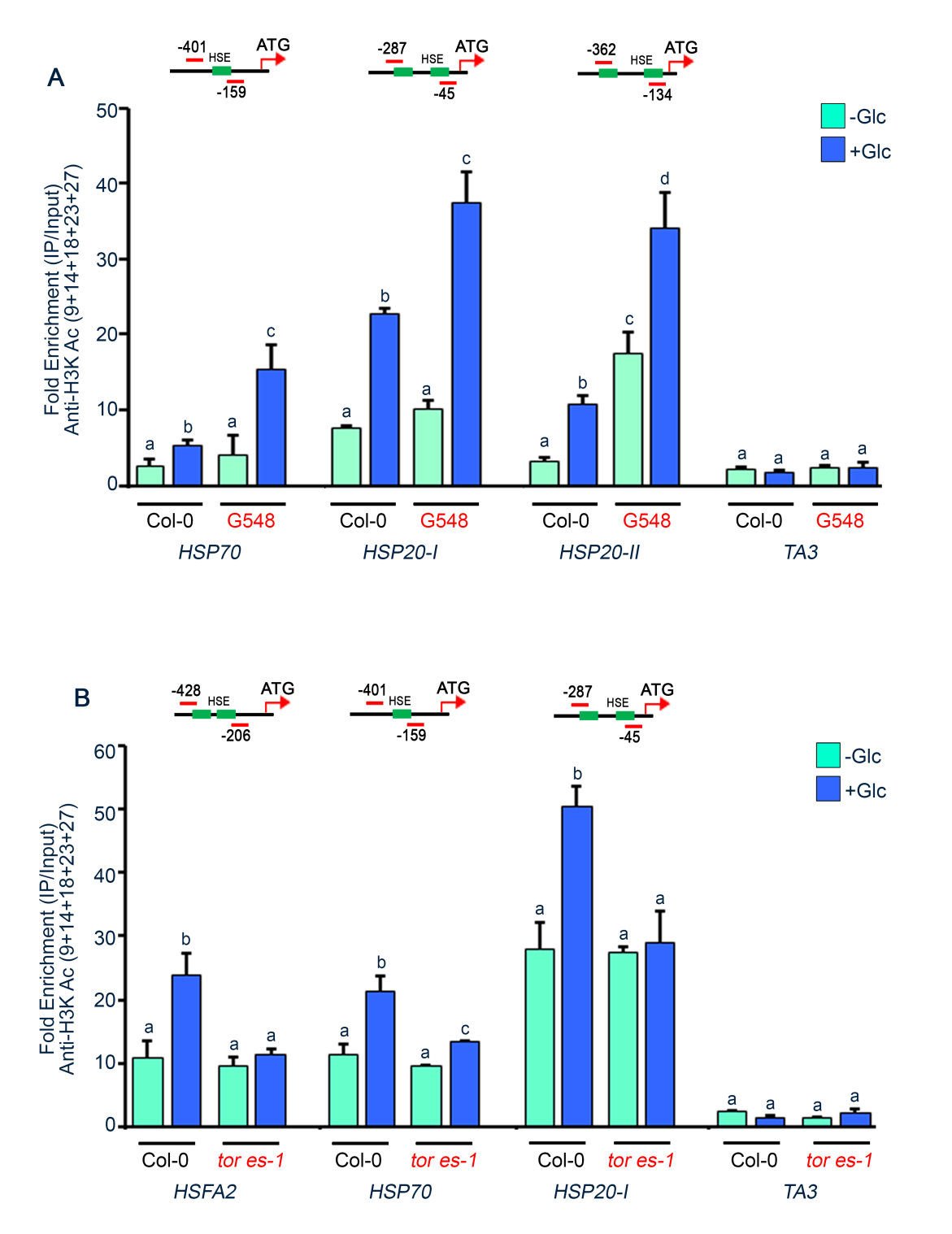


**Supplemental Figure S6. Second biological replicates showing enrichment of H3KAc on the promoters of HSP genes (related to Figure 5B and C) in TOR OE and RNAi lines. A,** ChIP-qPCR showing enrichment of histone H3K acetylation at the promoters of HS loci under Glc lacking/sufficiency in Col-0 and TOR overexpression line G548. **B,** ChIP-qPCR showing enrichment of histone H3K acetylation at the promoters of HS loci under Glc lacking/sufficiency in *tor-es1*. ChIP-qPCR analysis was performed on three technical replicates (n=3) from a single representative experiment. Experiment was independently repeated twice (biological replicates; n=2). Different letters between samples (Col-0 vs G548 and –Est vs +Est) in each gene denote statistical differences at P<0.05 as assessed by one-way ANOVA and Tukey’s HSD posthoc test.


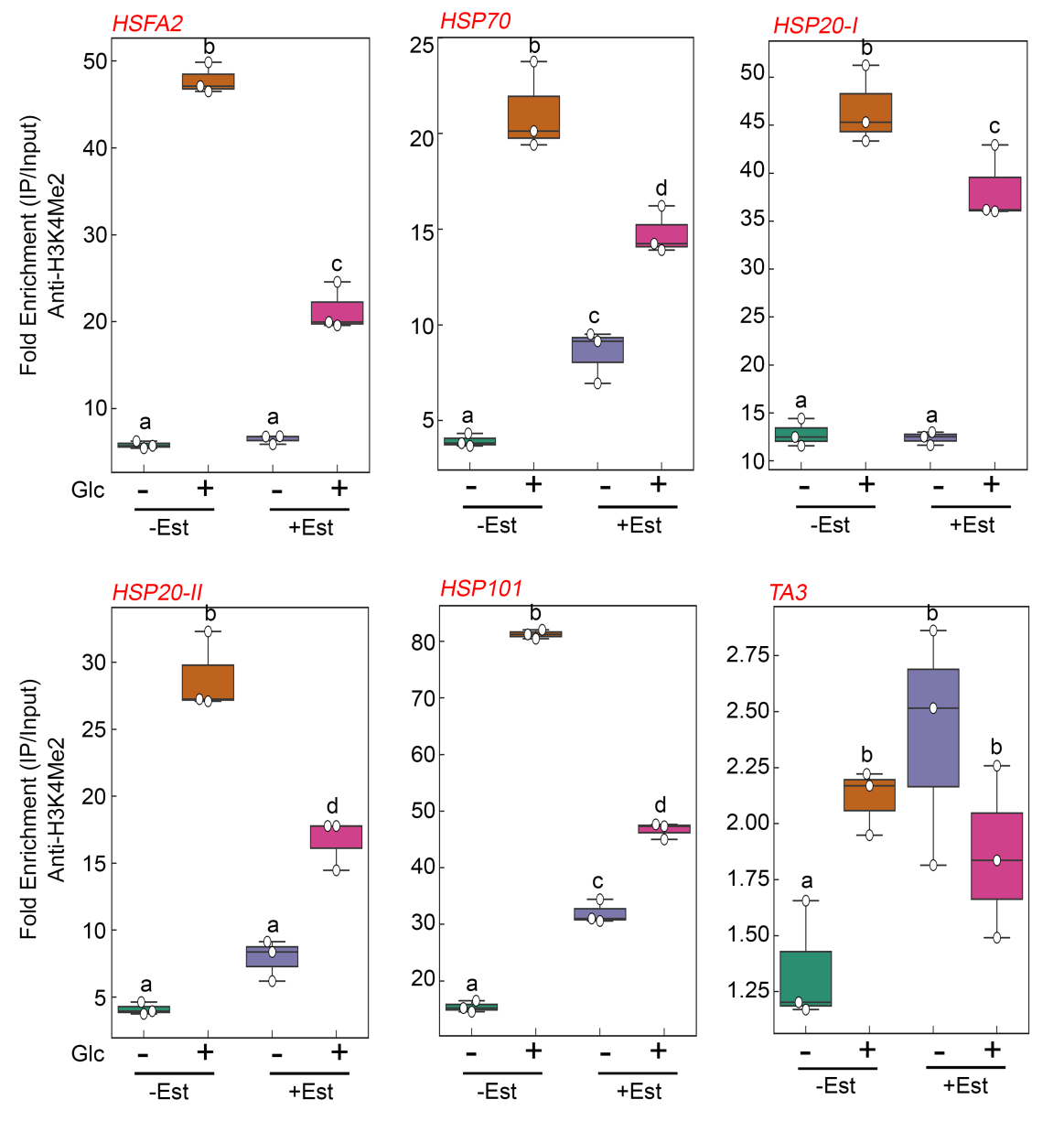


**Supplemental Figure S7. Glc through TOR induces enrichment of histone H3K4me2 marks at the promoters of HS genes.** ChIP-qPCR showing enrichment of histone H3K4me2 at the promoters of HS loci under Glc lacking/sufficiency. Promoter fragments containing cis-acting HSEs were immuno-precipitated using an anti-H3K4me2 antibody. The amount of immuno-precipitated promoter DNA was calculated by comparing samples treated without or with anti-H3K4me2 antibody. Ct values without and with antibody samples were normalized by input control. TA3 is a highly heterochromatinized DNA and was used as a negative control. Five days old *tor-es1* seedlings were transferred to 0.5X MS medium containing 20 µM β-estradiol for four days. Following estradiol treatment, seedlings were subjected to 24h energy starvation in MS medium without Glc and then supplied with 3h Glc (167 mM) treatment. For mock treatment, seedlings were transferred to an equal volume of DMSO (as used for estradiol) containing MS medium. ChIP-qPCR analysis was performed on three technical replicates (n=3) from a single representative experiment. Experiment was independently repeated twice (biological replicates; n=2). Different letters denote statistical differences at P<0.05 as assessed by one-way ANOVA and Tukey’s HSD posthoc test.


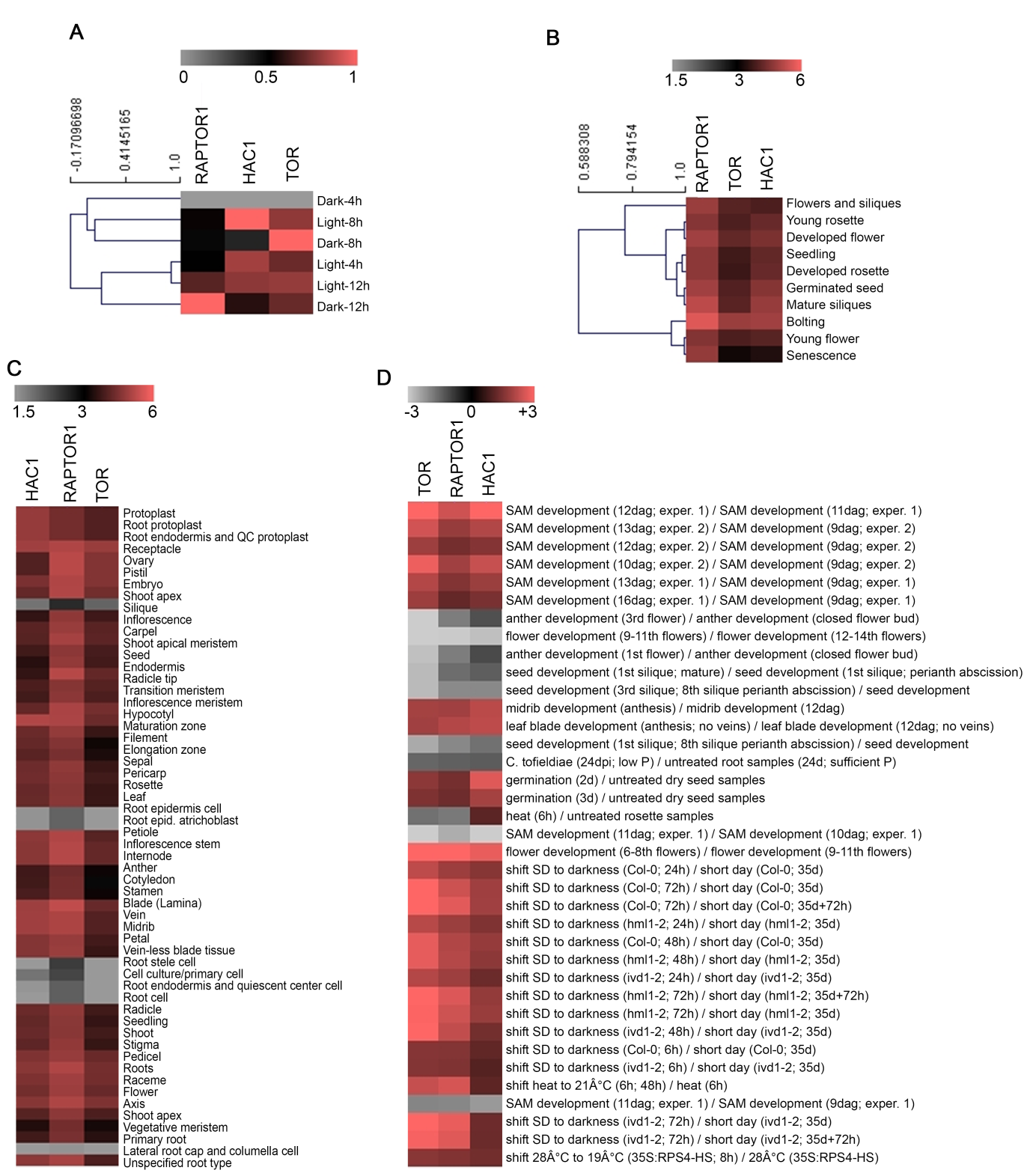


**Supplemental Figure S8. Diurnal light-dark cycle and Genevestigator transcriptome comparison show similarity in expression between TOR, RAPTOR1 and HAC1. A,** Heat map showing expression profiling of TOR, RAPTOR1 and HAC1. in diurnal light-dark transcriptome data obtained from public resources (Ferrari *et al.* 2019). **B-D,** Heat maps showing expression profiling of TOR, RAPTOR1 and HAC1 in anatomy, development and perturbations data obtained from Genevestigator.


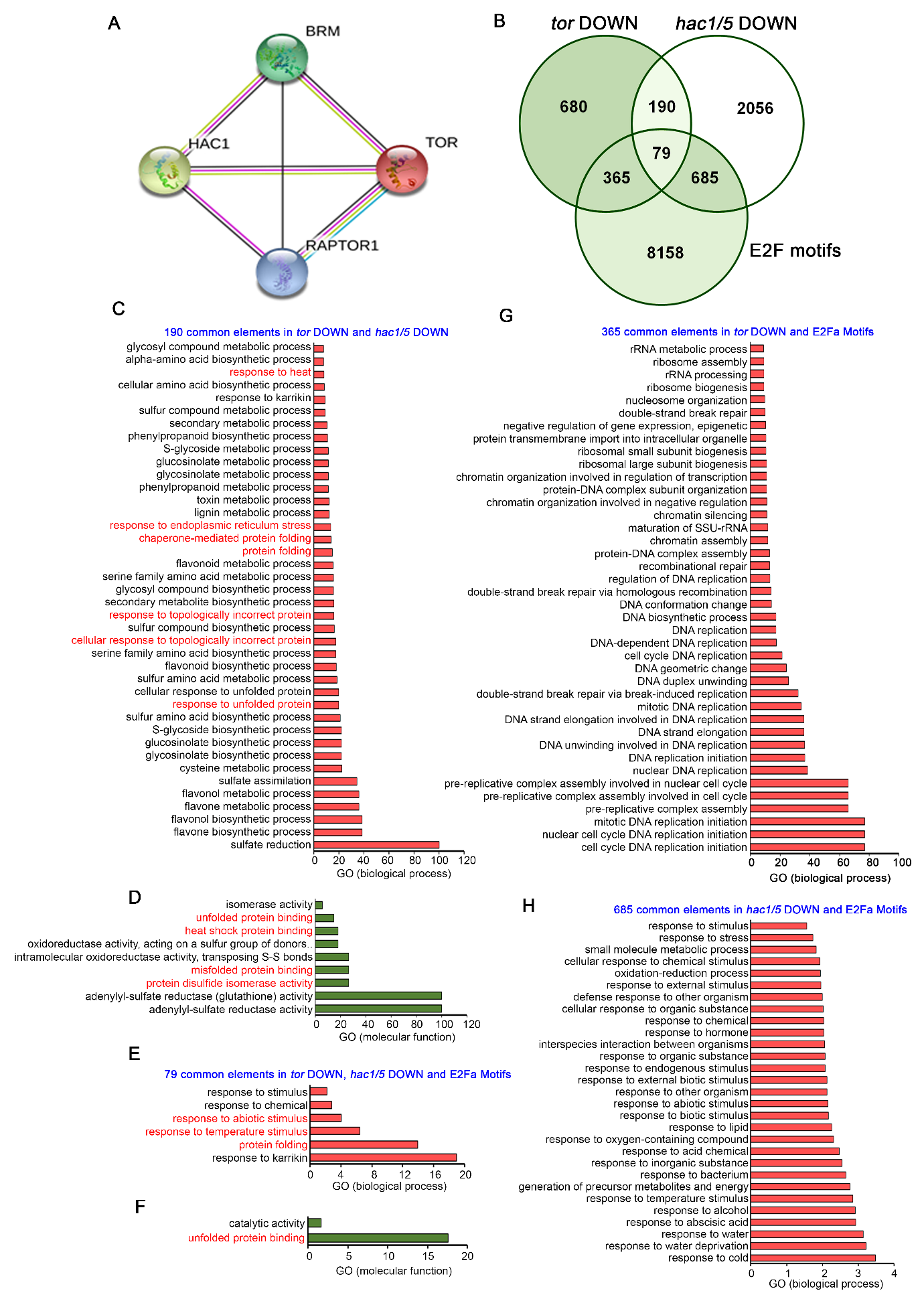


**Supplemental Figure S9. Protein-Protein interaction (PPI) between HAC1 and TOR complexes.** String data showing evidence of co-expression and interaction between TOR, RAPTOR1 and HAC1. Protein-Protein interaction (PPI) between HAC1 and TOR complexes was explored in String v.11 (Szklarczyk *et al.* 2019) which uses evidence from homologs in other species.


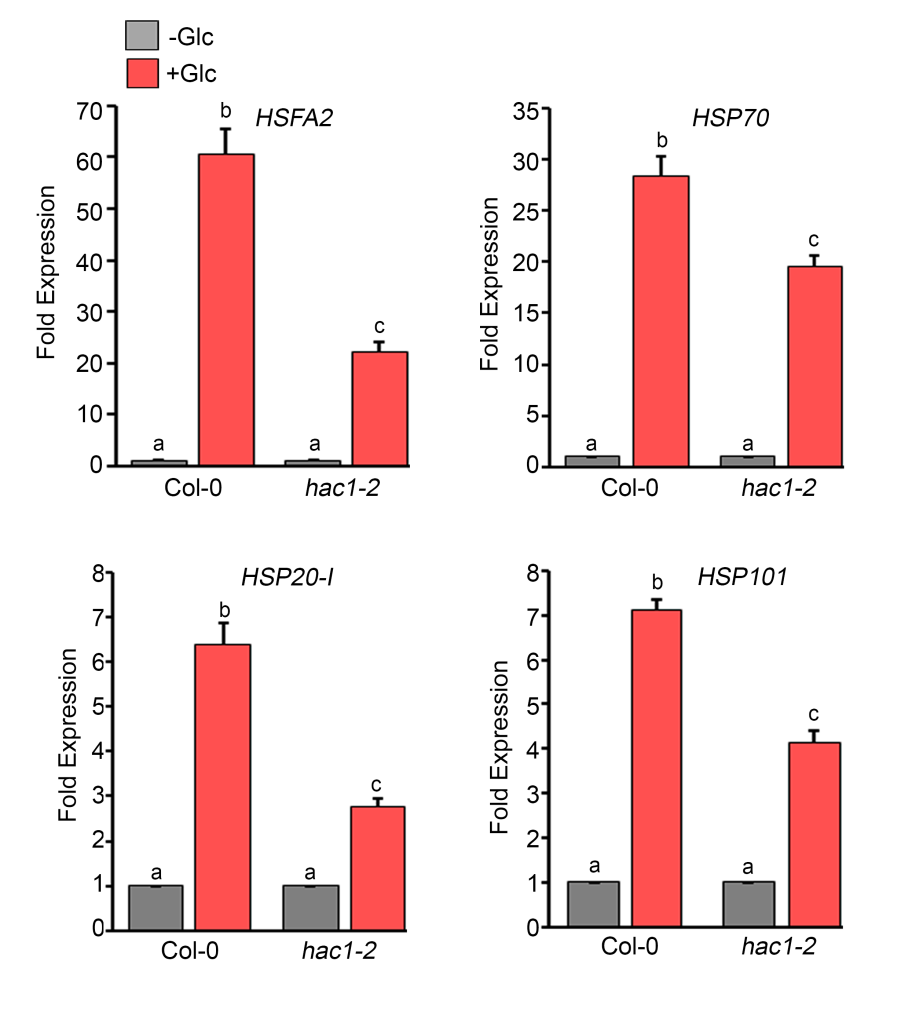


**Supplemental Figure S10. HAC1 promotes the expression of HSP genes under sugar sufficiency.** RT-qPCR expression of HS genes in Col-0 and *hac1-2* mutants in response to Glc (167 mM). RT-qPCR analysis were performed on three independent biological replicates (n=3). Bar plots represent mean values and error bars denote SE. Different letters denote statistical differences at P<0.05 as assessed by one-way ANOVA and Tukey’s HSD posthoc test.


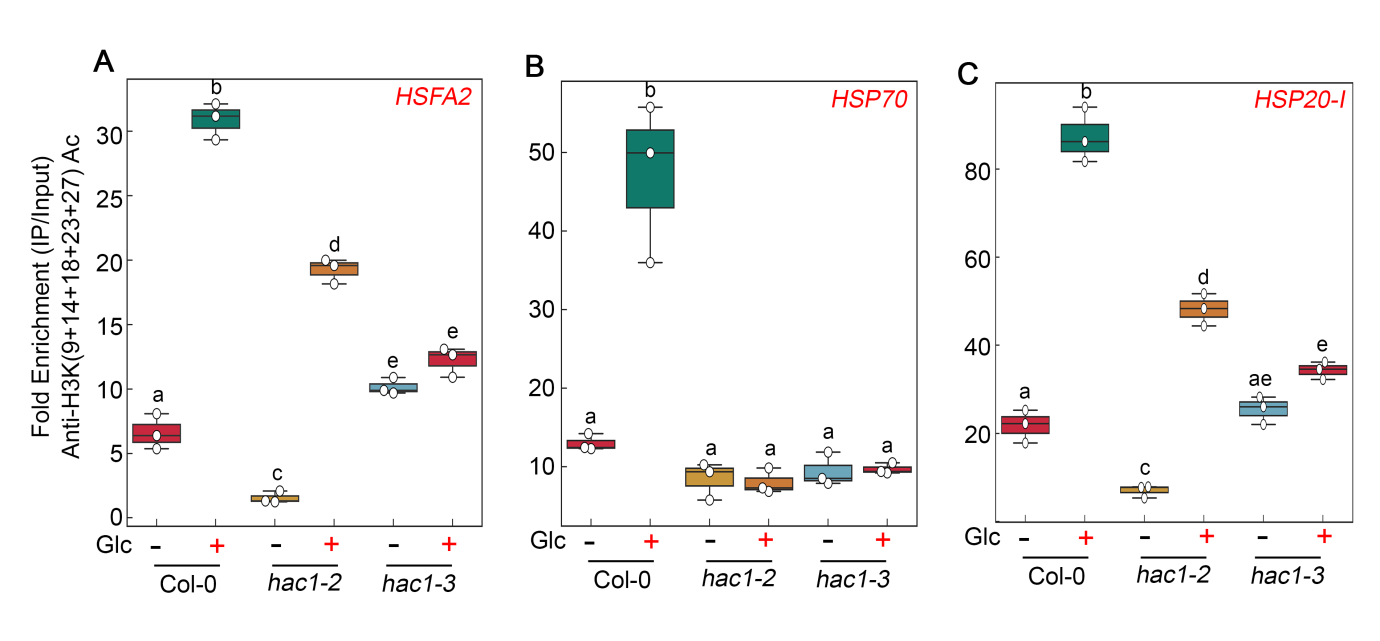


**Supplemental Figure S11. Second biological replicate showing enrichment of H3KAc on the promoters of HSP genes (related to Figure 6D) in Col-0 and *hac1-2* under Glc sufficiency. A-C,** ChIP-qPCR showing enrichment of histone H3K Ac (9+14+18+ 23+27) at the promoters of HS gene encompassing HSEs in Col-0 and *hac1* mutants. seven-day-old 0.5X MS grown Col-0 and *hac1* seedlings were subjected to 24h Glc starvation in MS medium without Glc and then supplied with 3h Glc (167 mM) treatment. ChIP-qPCR analysis was performed on three technical replicates (n=3) from a single representative experiment. Experiment was independently repeated twice (biological replicates; n=2). Different letters denote statistical differences at P<0.05 as assessed by one-way ANOVA and Tukey’s HSD posthoc test.


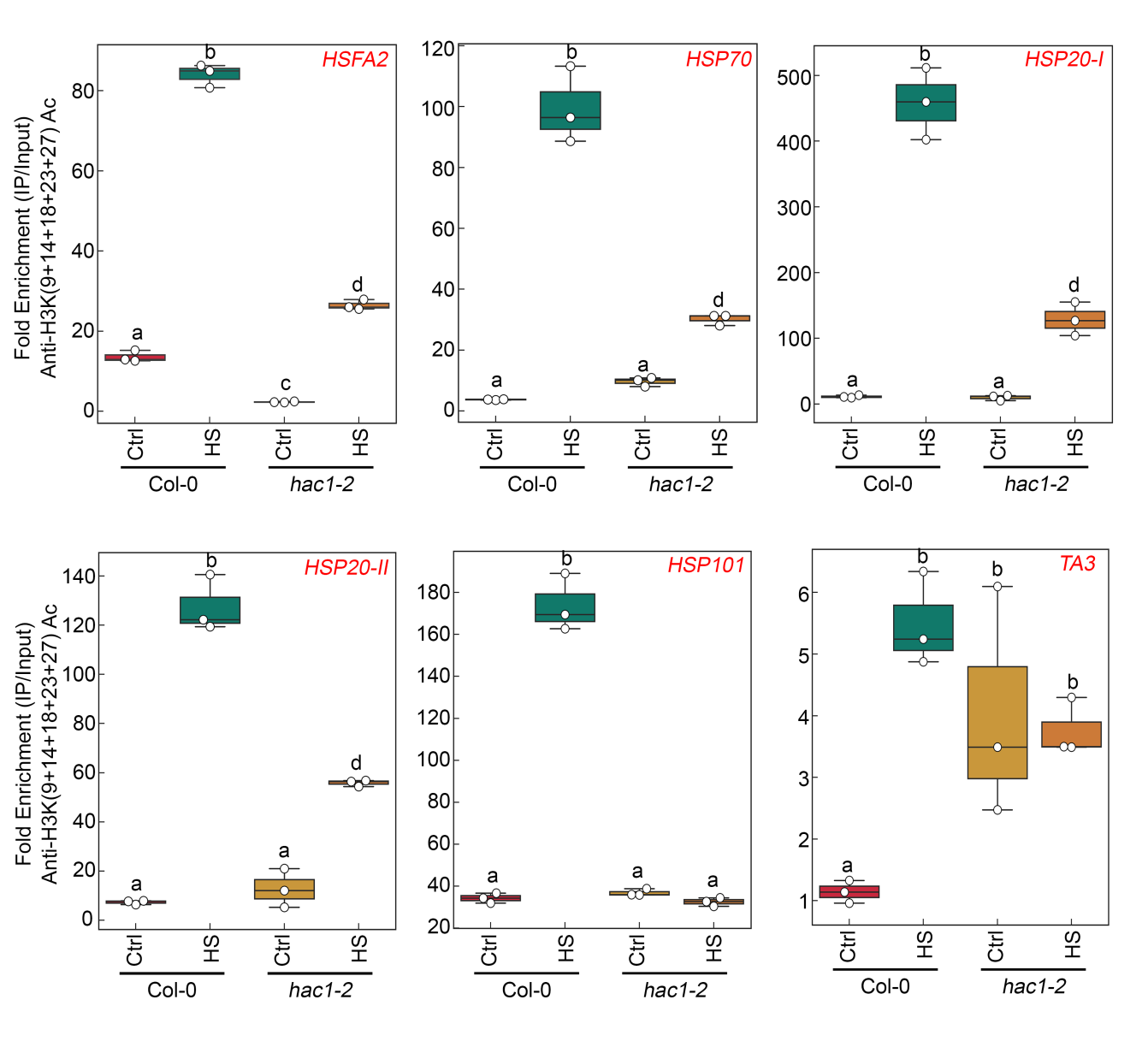


**Supplemental Figure S12. Second biological replicate showing enrichment of H3KAc on the promoters of HSP genes (related to Figure 7C) in Col-0 and *hac1-2* in response to heat stress.** ChIP-qPCR showing enrichment of histone H3K Ac (9+14+18+ 23+27) at the promoters of HS gene in Col-0 and *hac1-2* in response to heat stress. Seven-day-old 0.5X MS grown Col-0 and hac1-2 seedlings were subjected to 3h of HS treatment at 37°C. ChIP-qPCR analysis was performed on three technical replicates (n=3) from a single representative experiment. Experiment was independently repeated twice (biological replicates; n=2). Different letters denote statistical differences at P<0.05 as assessed by one-way ANOVA and Tukey’s HSD posthoc test.

**References**

Ferrari C., Proost S., Janowski M., Becker J., Nikoloski Z., Bhattacharya D., … Mutwil M. (2019) Kingdom-wide comparison reveals the evolution of diurnal gene expression in Archaeplastida. *Nature Communications* **10**.

Szklarczyk D., Gable A.L., Lyon D., Junge A., Wyder S., Huerta-Cepas J., … Von Mering C. (2019) STRING v11: Protein-protein association networks with increased coverage, supporting functional discovery in genome-wide experimental datasets. *Nucleic Acids Research* **47**, D607–D613.

Xiong Y., McCormack M., Li L., Hall Q., Xiang C. & Sheen J. (2013) Glucose-TOR signalling reprograms the transcriptome and activates meristems. *Nature* **496**, 181–6.
